# Efficient experimental quantum fingerprinting with channel multiplexing and simultaneous detection

**DOI:** 10.1038/s41467-021-24745-x

**Published:** 2021-07-22

**Authors:** Xiaoqing Zhong, Feihu Xu, Hoi-Kwong Lo, Li Qian

**Affiliations:** 1 Center for Quantum Information and Quantum Control, Dept. of Physics, University of Toronto, Toronto, Ontario Canada; 2 Hefei National Laboratory for Physical Sciences at the Microscale and Department of Modern Physics, University of Science and Technology of China, Hefei, China; 3 Center for Quantum Information and Quantum Control, Dept. of Electrical & Computer Engineering, University of Toronto, Toronto, Ontario Canada; 4 Department of Physics, University of Hong Kong, Hong Kong, China

**Keywords:** Quantum optics, Quantum information

## Abstract

Quantum communication complexity explores the minimum amount of communication required to achieve certain tasks using quantum states. One representative example is quantum fingerprinting, in which the minimum amount of communication could be exponentially smaller than the classical fingerprinting. Here, we propose a quantum fingerprinting protocol where coherent states and channel multiplexing are used, with simultaneous detection of signals carried by multiple channels. Compared with an existing coherent quantum fingerprinting protocol, our protocol could consistently reduce communication time and the amount of communication by orders of magnitude by increasing the number of channels. Our proposed protocol can even beat the classical limit without using superconducting-nanowire single photon detectors. We also report a proof-of-concept experimental demonstration with six wavelength channels to validate the advantage of our protocol in the amount of communication. The experimental results clearly prove that our protocol not only surpasses the best-known classical protocol, but also remarkably outperforms the existing coherent quantum fingerprinting protocol.

## Abstract (web summary)

Quantum fingerprinting could allow an exponential quantum advantage in a cryptographic protocol, but current schemes are still difficult to scale. Here, the authors exploit wavelength division multiplexing to increase the channel capacity and reduce the communication time without the need for demultiplexing.

## Introduction

Quantum communication is the study of information-transmission tasks that can be facilitated by using quantum mechanical systems^1^. The power of quantum mechanics enables quantum communication to perform tasks that could not be accomplished in a classical system. One of the best-known examples is quantum cryptography that enables information-theoretically secure communication between two parties that share random keys through quantum key distribution (QKD)^2–6^. Apart from quantum cryptography, quantum communication complexity (QCC)^7–10^ is another important example that shows quantum superiority over its classical counterpart–classical communication complexity^11–14^. In the basic model of communication complexity^13^, Alice and Bob each is given an n-bit string *x* and *y*, respectively. The classical communication complexity exploits the minimum amount of communication necessary among participants, namely the minimum number of bits of communication, such that they could compute a certain function *f*(*x*, *y*) correctly. This exploitation of a minimum amount of communication provides a lower bound for many related research areas, such as the study of VLSI circuit design, data structure and computer networks^13,14^. In the quantum version of communication complexity, the involved participants are allowed to communicate with quantum states instead of classical bits and QCC is then defined as the minimum number of qubits of communication exchanged between Alice and Bob^10^. It has been proven that, by using quantum superposition or entanglement, many quantum protocols of communication complexity are more efficient, that is, they require less communication (fewer qubits) than their classical counterparts^15–20^.

One remarkable protocol in QCC is quantum fingerprinting (QF)^21,22^ where quantum mechanics can help reducing the communication complexity exponentially compared with the classical case. In the fingerprinting mechanism, the simultaneous message-passing model is considered^11^. In this particular model, Alice and Bob have no shared randomness and are not allowed to communicate with each other. But they want to determine whether their inputs *x* and *y* are the same or different. In this case, a third party, the referee (Charlie), is involved and will solve this equality problem based on the inputs’ fingerprints that Alice and Bob send to her. The communication complexity in this model is defined as the amount of information communicated between Alice (Bob) and Charlie, which is equivalent to the minimum length of the fingerprints. Note that QF protocol is not concerned with communication security. It has been proven that, without any correlations or entanglement shared among the parties, quantum fingerprints require $O({{{{\rm{log}}}}}_{2}n)$ qubits^21^, which are exponentially smaller than the classical case where $O(\sqrt{n})$ bits are required^23–25^. To experimentally verify the advantage of quantum fingerprinting in small instances, a single-qubit fingerprinting protocol^26^ has been experimentally demonstrated in refs. ^27,28^ and has been shown to outperform the classical one-bit fingerprinting protocol. However, to demonstrate the exponential advantage of quantum fingerprinting, one must create fingerprints consisting of highly entangled qubit states^21^, which are beyond the reach of current technology. In ref. ^29^, a more practical QF protocol has been proposed and coherent states are used to construct the fingerprint. The minimum amount of communication required in this protocol is proven to be

1 $$Q=O(\mu {{{{\rm{log}}}}}_{2}n).$$

For simplicity, we call this coherent quantum fingerprinting protocol as CQF protocol in this letter. The total mean photon number of the fingerprint in this coherent quantum fingerprinting (CQF) protocol is *μ*. Therefore, for CQF protocol with a fixed *μ*, the minimum amount of communication can still be exponentially smaller than the classical fingerprinting protocol. Refs. ^30,31^ have successfully demonstrated the proof-of-principle experiment of CQF protocol and prove that less information is communicated in the CQF system compared with the best-known classical protocol^25^. Nonetheless, this CQF protocol uses a number of optical modes that is proportional to the input size *n*, hence the communication time is quadratically increased compared with the classical system^30,31^. In addition, the minimum amount of communication in CQF protocol has a dependence on *μ*, a value that has a lower limit due to experimental imperfections^29^, among which the dark counts from the single-photon detector (SPD) (used for Charlie’s detection) are a dominant factor^30^. As indicated in ref. ^31^ where superconducting-nanowire single-photon detectors (SNSPD) with very low dark count rates (<0.1 Hz) are applied, the performance of the CQF system is significantly improved and can even beat the classical limit. (Here, the classical limit refers to the lower bound of the amount of communication in any classical fingerprinting system. In our work, the lower bound given in Ref. ^31^ is used.) However, such SNSPDs are costly and will be impractical to implement on a large scale.

In this work, we propose a fingerprinting protocol utilizing the wavelength-division multiplexing (WDM) to reduce the communication time and improve the performance of CQF protocol. We call this protocol the WDM–CQF protocol. As a mature technique, WDM has been widely employed in classical communication systems to broaden the communication bandwidth and improve the communication efficiency^32,33^. It is natural to extend such an advantage into quantum communication systems. A lot of applications of WDM in quantum communication focus on providing shared infrastructure for both classical and quantum communication. There have been few studies using WDM to enlarge the quantum channel capacity^34–36^. Especially in ref. ^35^, coherent-state fingerprints are also used to study another QCC protocol–Euclidean problem, which aims at calculating the Euclidean distance of two real vectors of Alice and Bob. The authors similarly propose to employ multiplexing technique to improve the communication efficiency. However, demultiplexing followed by multiple individual detection systems are always needed in these studies. In this paper, WDM is used to increase the quantum channel capacity to reduce the communication time needed in the original CQF protocol without demultiplexing. All the quantum channels share the same detection system. More importantly, by removing demultiplexing and detecting the signals from different wavelength channels simultaneously, we can lower the value of *μ* in Eq. (1), thus reducing the amount of communication required in the original CQF protocol. With a large number of wavelength channels, it is in principle possible for our scheme to beat the classical limit without using SNSPDs. Here, we also report a proof-of-principle experimental implementation of the WDM–CQF protocol with six wavelength channels over 40-km fibers. Because of the use of time multiplexing during the process of information encoding, our implementation does not strictly show the reduction of communication time (this will be discussed in detail later). But the experimental results successfully validate that, with WDM being applied and with demultiplexing being removed for simultaneous detection, our system not only transmits much less information than the best-known classical protocol, but also reduces the amount of information transmitted in the original CQF protocol by more than half for large inputs.

## Result

### WDM–CQF protocol

In the CQF protocol^29^, Alice first prepares her coherent fingerprint ${\left|\alpha \right\rangle }_{{{{\rm{A}}}}}$ as

2 $${\left|\alpha \right\rangle }_{{{{\rm{A}}}}}={\mathop{\otimes }_{i=1}^{m}}{\left|{(-1)}^{E{(x)}_{i}}\frac{\alpha }{\sqrt{m}}\right\rangle }_{i}.$$

Bob’s fingerprint ${\left|\alpha \right\rangle }_{{{{\rm{B}}}}}$ has the same expression as Eq. (2) with changing subscript A into B and changing input *x* into *y*. The m-bit strings *E*(*x*) and *E*(*y*) are the codewords of Alice and Bob, respectively, obtained by applying error-correction code (ECC) to the *n*-bit input strings *x* and *y*. The ECC has a code rate *c* ($=\frac{n}{m} < 1$) and a Hamming distance *δm*. The use of ECC guarantees that when Alice’s and Bob’s inputs are different, the minimum number of different bits of *E*(*x*) and *E*(*y*) is *δm*. The codewords contain the information of the original inputs and are encoded into the phase of the coherent states (either 0 or *π* phase is added to the states). As indicated in Eq. (2), this coherent fingerprint is made up of *m*-coherent states. The mean photon number of each state is $\frac{| \alpha {| }^{2}}{m}(=\frac{\mu }{m})$. Then Alice and Bob forward their fingerprints to Charlie’s station through two optical channels, where each pair of Alice’s and Bob’s coherent states interferes with each other and is detected by Charlie’s SPDs. At Alice’s and Bob’s encoders, the amount of information sent by Alice and Bob (to the recipient Charlie) is shown in ref. ^29^ to be

3 $$Q=O(\mu {{{{\rm{log}}}}}_{2}m)=O\left(\mu {{{{\rm{log}}}}}_{2}\left(\frac{n}{c}\right)\right).$$

Since Alice and Bob each send *m*-coherent states to Charlie, the communication time is proportional to the input size *n* (as *m* = *n*/*c*).

To implement WDM–CQF protocol, Alice and Bob only need to divide their coherent fingerprints into *k* subfingerprints. Each subfingerprint consists of *m*/*k*-coherent states and is described as

4 $${\left|\alpha \right\rangle }_{{{{\rm{A}}}},j}={\mathop{\otimes }^{m/k}_{i=1}}{\left|{(-1)}^{{E}_{j}{(x)}_{i}}\frac{\alpha }{\sqrt{m}}\right\rangle }_{i}.$$

**Fig. 1:**
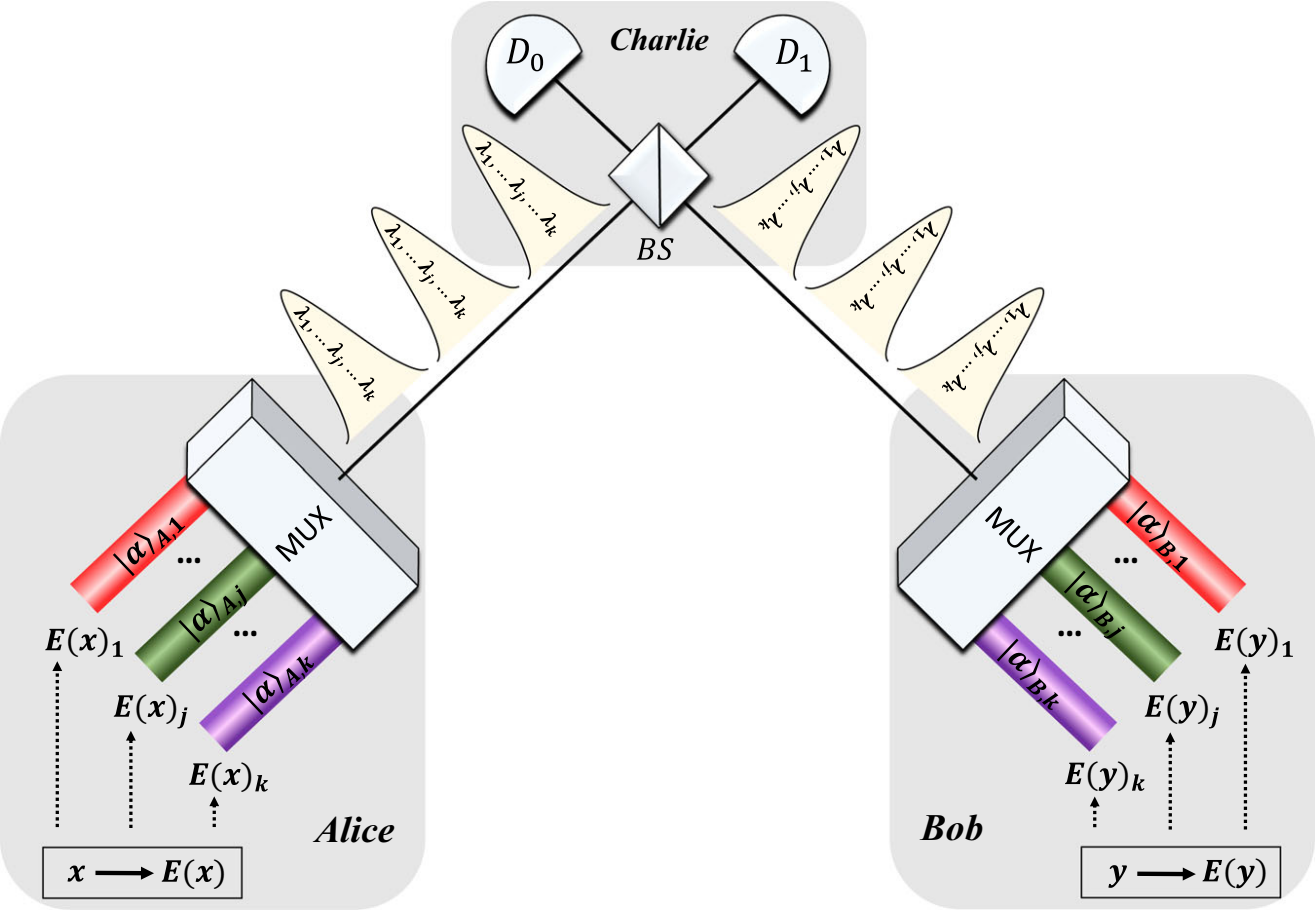
Theoretical scheme of coherent quantum fingerprinting with wavelength-division multiplexing. Alice (Bob) applies error-correction code to her (his) input *x*(*y*) and obtains *E*(*x*)(*E*(*y*)). Then she (he) divides *E*(*x*)(*E*(*y*)) into *k* subcodewords and prepares the corresponding subfingerprints *E*_*j*_(*x*)(*E*_*j*_(*y*)) in *k*-different wavelength channels. The *k* subfingerprints are multiplexed into one single-mode fiber through a multiplexer (MUX) and sent to Charlie’s beam splitter. On Charlie’s station, demultiplexing is not required. The *k* pairs of pulses interfere simultaneously and share a pair of single-photon detectors D_0_ and D_1_. Charlie records the total counts at D_0_ and D_1_, based on which Charlie determines whether the inputs are the same or different.

*E*_*j*_(*x*)_*i*_ is the *i*th bit of the *j*th subcodeword *E*_*j*_(*x*) (*j* ∈ [1, *k*]). Figure 1 shows the schematic set-up of the WDM–CQF protocol. As shown in Fig. 1, Alice and Bob assign each subfingerprint to a wavelength channel and multiplex the *k-*wavelength channels into a single optical channel. Then they send their fingerprints to Charlie for detection through the optical channels. In total, *m*/*k*-wavelength-composite pulses are sent from Alice/Bob to Charlie. On Charlie’s side, each pair of the wavelength-composite pulses interferes at the balanced beam splitter (BS) and is measured by two SPDs D_0_ and D_1_. Note that, the *k* pairs of coherent states at different wavelengths in each pulse interfere at the BS independently but simultaneously. Hence, the communication time is shortened to 1/*k* times of its original value. We remark that, all the wavelength channels share the same BS and SPDs, thus saving experimental components. Since the coherent fingerprints used in our protocol are the same as that in the original CQF protocol, the amount of information transmitted from Alice and Bob to Charlie is still $O(\mu {{{{\rm{log}}}}}_{2}m)$. In fact, except for adding the additional wavelength channels, the WDM–CQF system is very similar to the original CQF system. One could treat CQF protocol as a special case of WDM–CQF protocol with a single-wavelength channel (*k* = 1).

After the measurement, Charlie has to determine whether Alice’s and Bob’s inputs are the same or not by checking the total counts at D_0_ and D_1_. Ideally, if there is any count at D_1_, the inputs *x* and *y* should be different. This is because, if Alice and Bob have the same inputs, their coherent fingerprints are the same and the phase interference of the same states results in clicks only at D_0_. If the inputs are different, a portion of the interfering states have a *π*-phase difference. The photons in these states are registered at D_1_. However, experimental imperfections, such as dark counts of SPD, would also give clicks in D_1_ even when the inputs are the same. Here we adopt the decision mechanism introduced in ref. ^30^. In ref. ^30^, for the equal and different inputs cases, photon counts at detector D_1_ have the binomial distributions B(*m*, *P*_E_), and B(*m*, *P*_D_) respectively. *P*_E_ and *P*_D_ are the probabilities of D_1_ obtaining a click in a single-detection window. Based on these distributions, a threshold *C*_1,th_ is chosen. Charlie then compares the total counts at D_1_ with *C*_1,th_. If the total counts are smaller than *C*_1,th_, Charlie concludes that the inputs are equal. Otherwise, Charlie concludes that the inputs are different. In our WDM–CQF system, since each detection event is independent, the photon counts at D_1_ also have the binomial distributions B(*M*, *P*_E_) and B(*M*, *P*_D_). Note that *m* is replaced by *M*, since in total, *M*-wavelength-composite pulses are sent from Alice/Bob to Charlie in the WDM–CQF protocol. The amplitude of each pulse is

5 $$\frac{\mu }{m/k}=\frac{\mu }{M}.$$

The detection probabilities *P*_E_ and *P*_D_ are

6 $${P}_{{{{\rm{E}}}}}={P}_{{{{\rm{E}}}},{{{\rm{signal}}}}}+{P}_{{{{\rm{dark}}}}}=(1-\nu )(1-{e}^{-\frac{2\mu \eta }{M}})+{P}_{{{{\rm{dark}}}}},$$

7 $${P}_{{{{\rm{D}}}}} 	={P}_{{{{\rm{D}}}},{{{\rm{signal}}}}}+{P}_{{{{\rm{dark}}}}}\\ 	=(\delta \nu +(1-\delta )(1-\nu ))(1-{e}^{-\frac{2\mu \eta }{M}})+{P}_{{{{\rm{dark}}}}}.$$

For the probability *P*_D_, we assume the worst-case scenario that the codewords *E*(*x*) and *E*(*y*) have the minimum distance. *ν* is the interference visibility that considers the imperfect interference due to various factors. For example, the interfering states from Alice and Bob might not arrive at Charlie’s station simultaneously. Their polarization states might not be exactly the same after traveling a long distance and the phase drift could also be different. To maximize *ν*, one must minimize these mismatches. *η* is the optical channel transmittance. Here we consider that the optical channel loss between Alice and Charlie is the same as the loss between Bob and Charlie, i.e., *η*_A_ = *η*_B_ = *η*. If the channel losses are different, Alice and Bob simply use different signal intensities *μ*_A_ and *μ*_B_ such that *μ*_A_ × *η*_A_ = *μ*_B_ × *η*_B_. If the detector’s efficiency *η*_dec_ is taken into consideration, then *η* = *η*_A/B_ × *η*_dec_. *P*_dark_ is the dark-count probability per detection gate of the SPDs. The error probability for this decision mechanism is

8 $${P}_{{{{\rm{error}}}}}=\max [P({C}_{1,{{{\rm{E}}}}}\, > \,{C}_{1,{{{\rm{th}}}}}),P({C}_{1,{{{\rm{D}}}}}\, < \,{C}_{1,{{{\rm{th}}}}})],$$

and it should be smaller than the tolerable probability *ϵ*. *C*_1,E_ and *C*_1,D_ are the detected total counts at detector D_1_ for the equal and different input cases, respectively. For each input size *n*, the choice of threshold *C*_1,th_ depends on the total mean photon number *μ*. As indicated in Eq. (6) and Eq. (7), when *μ* is so small such that *P*_E/D,signal_ ≪ *P*_dark_, the probabilities *P*_E_ and *P*_D_ are dominated by *P*_dark_ and the distributions *B*(*m*/*k*, *P*_E_) and *B*(*m*/*k*, *P*_D_) are fairly close to each other. Consequently, the error probability would be very large. Therefore, a large value of *μ* is preferred for minimizing the error probability. However, as mentioned before, the amount of communication required by the coherent fingerprint is proportional to the mean photon number *μ*. So, for each input size *n*, one has to balance the two demands of low error probability and a small amount of communication. That is to say, one has to find the minimum *μ* (and its corresponding threshold *C*_1,th_), which gives the error probability *P*_error_ smaller than *ϵ*. More details about the optimization of *μ* can be found in “Method”.

It is straightforward to think that the lower the dark-count probability *P*_dark_ is, the smaller *μ* can be found. Ref. ^31^ uses SNSPDs with ultralow dark count rate (0.11 Hz) significantly reduces the value of *μ*, hence, it can beat the classical limit. However, SNSPDs are much more expensive than the regular SPDs and require very low temperature. Instead of using SNSPDs to decrease *P*_dark_, our WDM–CQF protocol simply increases the signal probability *P*_E/D,signal_ in Eq. (6) and Eq. (7) by simultaneously detecting *k* pairs of wavelength components. Because a low value of *μ* is preferred in the protocol, most of the coherent states are empty when they arrive at Charlie’s station. The signal probability *P*_E/D,signal_ primarily comes from the photons in one-wavelength component. With a reasonable number of wavelength channels (say 1 ≤ *k* ≤ 1000), the probability of more than one-wavelength component containing photons when arriving at Charlie’s station is so low (even lower than *P*_dark_) that we can simply ignore the multi-wavelength contributions to the detection event (detailed analysis can be found in Methods) Moreover, the information of which wavelength component carries a photon is not important and only the total counts detected on D_1_ are valued. Therefore, demultiplexing is not necessary on Charlie’s station. *k* pairs of coherent states at different wavelengths interfere simultaneously and are detected by a single pair of detectors in each detection window. Compared with the single-wavelength CQF protocol, the signal probability *P*_E/D,signal_ is increased without changing *μ* and the error *P*_error_ is then decreased. Therefore, to achieve the same tolerable error probability *ϵ*, our WDM–CQF protocol requires a lower value of *μ* than the single-wavelength CQF protocol. Consequently, as indicated in Eq. (3), less amount of communication is required in our WDM–CQF protocol than the single-wavelength CQF protocol. More wavelength channels are used, less *μ* is needed, and greater gain of the amount of communication is obtained by our protocol.

**Fig. 2:**
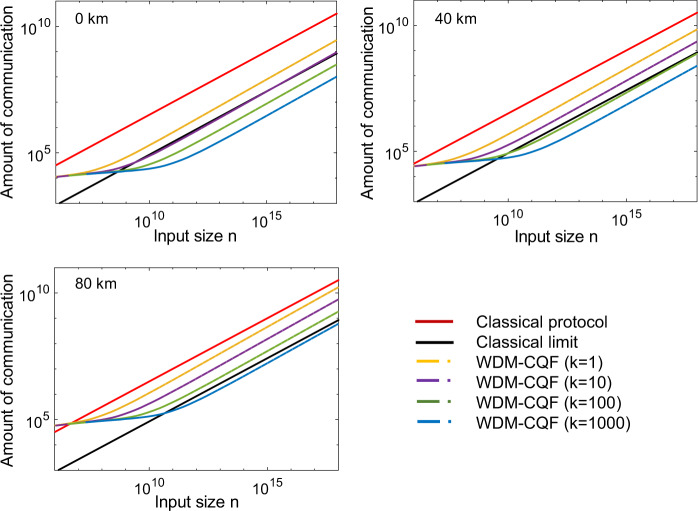
Log–log plot of the simulation results of the amount of communication required in different fingerprinting protocols as a function of input size *n* for three distances, 0 km, 40 km, and 80 km. The solid red line represents the best-known classical fingerprinting protocol^25^. The solid black line represents the classical limit introduced in^31^. The dash curves are to the simulation results of coherent quantum fingerprinting (CQF) protocol with wavelength-division multiplexing (WDM). Different values of *k* correspond to a different number of wavelength channels. When *k* = 1, the scheme becomes the original CQF protocol. In the simulation, we use parameters achievable with single-photon avalanche diodes, with a dark-count rate of 100 Hz and 25% detector efficiency. The interference visibility is assumed to be 97% and the detection window is 500 ps. The applied error-correction code has a code rate *c* = 0.2398 and *δ* = 0.22. The total mean photon number *μ* for each *n* and *k* is optimized to fulfill the condition *P*_error_ < *ϵ* = 10^−5^.

Figure 2 shows the amount of communication between Alice/Bob and Charlie over 0 km, 40 km, and 80 km fibers in different fingerprinting protocols as a function of the input size *n*. Note that the distance considered in the work is the total length of fibers that connect between Alice and Charlie and fibers that connect between Bob and Charlie. In short, we just call it the overall distance between Alice and Bob. In this log–log plot, practical experimental parameters are considered. The interference visibility is assumed to be 97%. The dark-count rate and the detector’s efficiency are 100 Hz and 25%, respectively. (We use the parameters from the best available commercial SPD ID230 from ID Quantique to show the best performance of our protocol.) The detection window is 500 ps. The tolerable error probability is chosen to be *ϵ* = 10^−5^. The input size *n* varies from 10^5^ to 10^18^. (Details about the simulation are discussed in Methods.) As shown in Fig. 2, for different distances, all the WDM–CQF protocols require less communication than the best-known classical fingerprinting protocol. As *k* gets larger, the advantage of WDM–CQF protocol is more evident. Compared with the original CQF protocol (*k* = 1), our WDM–CQF protocol with *k*≥100 reduces the amount of communication by at least one order of magnitude. In fact, with the parameters used in this simulation, the original CQF protocol (*k* = 1) cannot beat the classical limit even when the overall distance between Alice and Bob is 0 km. However, with only *k* = 10 wavelength channels applied, our WDM–CQF protocol can transmit less information than the classical limit for 0 km. When the distance increases, more photons are needed to compensate the channel loss. Hence, the amount of communication in the coherent fingerprinting system increases with the channel distance. But, in our WDM–CQF protocol, the channel loss can be compensated by adding wavelength channels. Therefore, even when the distance increases, our WDM–CQF protocol can always beat the classical limit without using SNSPDs, as depicted in Fig. 2. Remarkably, when the overall distance between Alice and Bob is 40 km, our WDM–CQF protocol requires around 100-wavelength channels to beat the limit. We remark that it is currently feasible to achieve around 100 simultaneous channels by using WDM, since there have been many reports of classical transmission experiments with WDM over more than 100 channels^37,38^. Moreover, the total cost of adding wavelength channels is much lower compared with applying SNSPDs. When the distance is longer, more channels are required by our WDM–CQF protocol to beat the classical limit. The implementation of WDM–CQF with a large number of wavelength channels is very challenging. To circumvent this issue, one can combine other multiplexing schemes with wavelength multiplexing to reduce the number of wavelength channels. For example, one can use time-division multiplexing (TDM), i.e., use fast modulators to add more temporal channels within one detection window. We would like to emphasize that our WDM–CQF protocol takes the advantage of the simultaneous detection of many bits of information within one detection window. These bits of information can be distributed in, but not confined to the wavelength channels.

### Experimental set-up

**Fig. 3:**
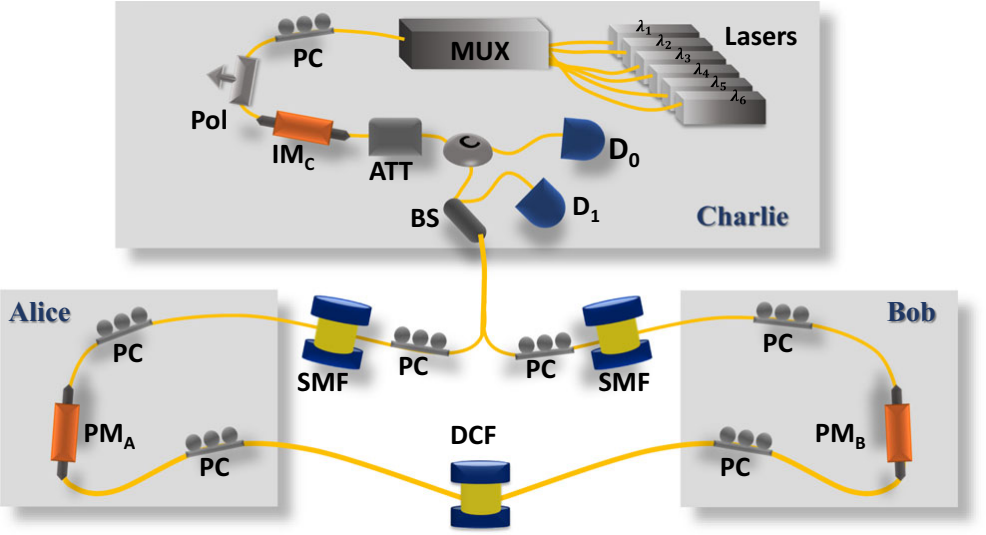
Schematic experimental set-up of coherent quantum fingerprinting with wavelength-division multiplexing. Six continuous-wave lasers are located on Charlie’s side with wavelength ranging from 1542.9 nm to 1554.9 nm, equally spaced by *δλ* = 2.4 nm. Photons coming out the lasers are multiplexed through a multiplexer (Mux) into a single-mode fiber and pass through a polarizer (Pol). An intensity modulator (IM) and an optical variable attenuator are used to create weak coherent pulses. The pulses then enter the loop through a circulator (C) and a beam splitter (BS) and travel to Alice/Bob through 20 km single-mode fibers (SMF). Alice and Bob are separated by another 6.9 km of compensation-dispersion fibers (DCF). On Alice’s (Bob’s) station, the phase modulator (PM) is on only when the clockwise (counterclockwise) traveling pulses arrive and the phase information is added to the pulses accordingly. After the phase modulation, the clockwise and counterclockwise traveling pulses go back to Charlie and interfere with each other at Charlie’s beam splitter. The results are recorded by two single-photon detectors D_0_ and D_1_. Polarization controllers (PC) are designed for the polarization alignment for the beams in six-wavelength channels.

In this section, we show a proof-of-concept experimental demonstration of our WDM–CQF protocol. Six-wavelength channels are used and a two-way quantum communication system consisting of a Sagnac interferometer is employed. This system configuration is similar to that of a twin-field QKD system^39^. The Sagnac arrangement is chosen to provide a phase reference between Alice and Bob. Moreover, the common path feature of Sagnac interferometer automatically stabilizes the phase fluctuation along the optical channel and ensures that two beams emerge at the beam splitter simultaneously. The schematic experimental set-up is shown in Fig. 3. On Charlie’s station, the continuous waves (cw) coming out of six laser modules (PRO 800, wavelength $\lambda \in \left\{1542.9,1545.3,1547.7,1550.1,1552.5,1554.9\right\}$ nm) are multiplexed into a single-mode fiber (SMF) through a multiplexer (Jobin Yvon-Spex, Stimax WDM, 100 GHz) and are forwarded to Charlie’s intensity modulator (IM_C_) through a polarizer. The output power of each laser module is individually adjusted such that the signal in each wavelength channel has the same intensity. IM_C_ is used together with an optical attenuator (Att_C_) to create weak wavelength-composite pulses (500 ps pulse width) at a repetition rate of 50 MHz. Then Charlie sends the pulses to Alice and Bob through an optical circulator and a 50:50 fiber-based beam splitter (BS). After passing through the BS, the pulses split into clockwise and counterclockwise traveling beams and travel through a 20-km single-mode fiber spool SMF_B_ or SMF_A_, respectively. When the clockwise (counterclockwise) traveling pulses arrive at Bob’s (Alice’s) station, the fiber-based phase modulator PM_B_ (PM_A_) is turned off and no information is encoded into the pulses. This is important because it guarantees that no information is communicated between Alice and Bob, even though they are connected directly through fibers. We remark that security is not a concern in quantum fingerprinting, since the main purpose is to reduce communication complexity. Then the clockwise (counterclockwise) traveling pulses go through 6.9 km dispersion-compensation fibers (DCF) before arriving at Alice’s (Bob’s) station. Note that this 6.9 km DCF is designed for temporal dispersion. In our system, each pulse created by Charlie has six-wavelength components. To modulate the phase of each component individually, we use the natural property of fiber, that is chromatic dispersion, to separate these wavelength components in time. The dispersion parameters (around 1550 nm) of the SMF and DCF in our set-up are *D*_SMF_ = 17 ps/(nm ⋅ km) and *D*_DCF_ = −99 ps/(nm ⋅ km), respectively. Given that the wavelength difference between two adjacent modes is *δλ* = 2.4 nm, we can estimate the time difference of arrival *δT*_A/B_ at Alice’s/Bob’s station between the adjacent-wavelength components by

9 $$\delta {T}_{{{{\rm{A}}}}/{{{\rm{B}}}}}=| {D}_{{{{\rm{SMF}}}}}\times \delta \lambda \times {l}_{{{{{\rm{SMF}}}}}_{{{{\rm{B}}}}/{{{\rm{A}}}}}}+{D}_{{{{\rm{DCF}}}}}\times \delta \lambda \times {l}_{{{{\rm{DCF}}}}}| .$$

**Fig. 4:**
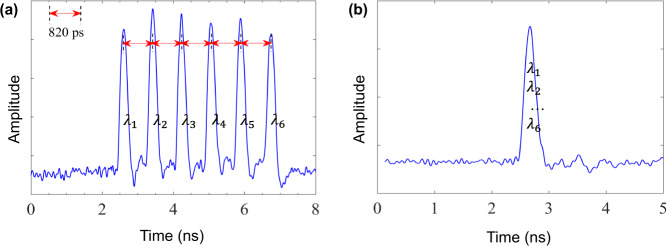
Arrival times of different wavelength components. The wavelength components traveling in counterclockwise direction are (**a**) temporally separated at Bob’s station for ease of individual modulation, while they are (**b**) combined into the same time slot at Charlie for detection. The components traveling in clockwise direction have the same time distribution since Alice’s and Bob’s station are symmetric. Note, one should ignore the slight amplitude difference between the channels as this plot is taken prior to amplitude fine adjustments.

*l*_SMF_ and *l*_DCF_ are the lengths of the single-mode fibers and dispersion-compensation fibers, respectively. Figure 4a shows the different arrival times of the six wavelength components at Bob’s station after traveling through 20 km SMFA and 6.9 km DCF. As indicated, *δT*_A/B_ in our experiment is around 820 ps, which enables Alice (Bob) to modulate the phases of the six-wavelength components sequentially by using an 800 ps phase-modulation window for each component. After the phase modulation, Alice (Bob) forwards the pulses to Charlie’s BS through another 20 km fiber spool SMF_A_ (SMF_B_). The length of the DCF is designed to ensure that the six-wavelength components overlap with each other in time and become a single-wavelength-composite pulse at Charlie’s BS. The time difference of arrival at Charlie’s station can be estimated by

10 $$\delta {T}_{{{{\rm{C}}}}}=| {D}_{{{{\rm{SMF}}}}}\times \delta \lambda \times ({l}_{{{{{\rm{SMF}}}}}_{{{{\rm{A}}}}}}+{l}_{{{{{\rm{SMF}}}}}_{{{{\rm{B}}}}}})+{D}_{{{{\rm{DCF}}}}}\times \delta \lambda \times {l}_{{{{\rm{DCF}}}}}| ,$$

which is around 0 ps. As shown in Fig. 4b, after traveling through the whole loop, the six-wavelength components arrive at Charlie’s BS at the time and overlap with each other. The clockwise and counterclockwise traveling pulses interfere with each other at Charlie’s BS and are detected by two SPDs D_0_ and D_1_. We emphasize that demultiplexing is not needed on Charlie’s station. As mentioned before, the wavelength information of the detected photon is not important. Only the total counts at D_1_ determine Charlie’s output. Therefore, the six pairs of coherent states at different wavelengths share the same BS and detectors. The SPDs are commercial avalanche photodiodes (ID220) with an efficiency of 20% and a dark-count rate of 1000 Hz. The detection window is about 500 ps. After the measurement, Charlie counts the total number of click events in detector D_1_ only and compares it with a predetermined threshold value *C*_1,th_. If the number is smaller than the threshold *C*_1,th_, he announces that the inputs of Alice and Bob are equal. Otherwise, he concludes that the inputs are different.

The most challenging problem in our experiment is the wavelength-dependent polarization-mode dispersion of long optical fibers^40–42^. Due to the birefringence of the optical fiber, the polarization state varies along the fiber. To guarantee the high-interference visibility, the polarization of the interfering pulses should be aligned with each other. This alignment could be easily accomplished with one polarization controller (PC) if only one-wavelength channel is applied. However, the variation of polarization strongly depends on wavelength, especially for long fibers. Therefore, when multiple-wavelength channels are used, the polarization states at different wavelengths evolve differently, making the polarization alignment difficult. As a result, the interference visibility would be affected significantly. To solve this issue, we utilize the principal state of polarization (PSP)^41^. For a fiber system, there are always two orthogonal PSPs, the polarization evolution of which does not depend on the wavelength to the first order. That is to say, if the input polarization states of the different wavelength components are the same and aligned to the input psp of the optical fibers, the output polarization states should also be the same. Note that we ignore the high-order polarization-mode dispersion since that the wavelength range in our experiment is only 12 nm. In our setup, there are three long-fiber spools and two polarizers (integrated with the phase modulators) used in the Sagnac loop. Therefore, six polarization controllers are inserted into the Sagnac loop for the alignment, as shown in Fig. 3. One PC at one end of a fiber spool is designed to align the input-polarization state to the input PSP, the other PC at the other end is used to align the polarization state to the output PSP of the fibers. With such alignment scheme, we are able to maintain our interference visibility to be 97% over a 12 nm bandwidth.

Another challenge in our implementation is the calibration of the fiber length. First of all, as indicated in Eq. (9), the lengths of SMF and DCF determine the time difference of arrival *δT* among different wavelength components. On one hand, we must ensure that on Alice’s and Bob’s stations, *δT*_A/B_ is large enough such that Alice and Bob can modulate the different wavelength components separately, on the other hand, when the pulses travel back to Charlie’s station, *δT*_C_ should be 0 ps. Therefore, the fiber lengths of SMF_A_, SMF_B_, and DCF are carefully calibrated to fulfill these two conditions. Additionally, it is also crucial to ensure that the clockwise and counterclockwise traveling pulses should never “collide” at Alice’s and Bob’s phase modulators. This is because Alice (Bob) should only modulate the clockwise (counterclockwise) traveling pulses. To avoid the pulse collision, small segments of fibers can be added or deleted on Alice’s and Bob’s station. Meanwhile, all the phase and intensity modulators are driven and synchronized by a high-speed arbitrary-waveform generator (AWG, Keysight M8195A). The delays of Alice’s and Bob’s phase-modulation signals are well adjusted to ensure that the modulation signals only act on the intended pulses.

### Experimental result

The experiment was run over seven different values of the input size *n*, ranging from 1.4 × 10^6^ to 1.1 × 10^9^. For each input size *n*, we tested both the case where the inputs are the same (*x* = *y*) and the case where the inputs have one-bit difference (*x* ≠ *y*, *E*(*x*) and *E*(*y*) have (*δm*)-bit difference). *δ* = 0.22 and the code rate *c* = 0.2398. The total photon numbers sent out by Alice (*μ*_*A*_) and Bob (*μ*_*B*_) for different input sizes are listed in Table 1. The reason why Alice and Bob have different *μ* is that the channel loss between Alice and Charlie is slightly different from the loss between Bob and Charlie. Based on the average photon numbers reported, we can determine the threshold value of the total counts *C*_1,th_ at detector D_1_ as well as its corresponding error probability *P*_error_. Note that the total mean photon numbers in this implementation are close to but not exactly the optimal values. Therefore, the error probabilities for some cases are larger than *ϵ* = 10^−5^. Nevertheless, the largest *P*_error_ is 2.7 × 10^−5^ that is tolerable^30^. The total counts recorded by detector D_1_ for the equal-input case (*C*_1,E_) and the different input cases (*C*_1,D_) are also listed in Table 1. For all the seven different input sizes, Charlie could successfully differentiate between the equal and different inputs by comparing the total counts at D_1_ with the threshold *C*_1,th_. *Q* is the total amount of information that has been transmitted to Charlie by Alice and Bob. It is calculated as the equivalent number of qubits that has been transmitted. To show the advantage of our WDM–CQF protocol, we calculated the ratio ${\gamma }_{{{{\rm{C}}}}}=32\sqrt{n}/Q$ ($32\sqrt{n}$ is the minimum amount of communication required in the best-known classical fingerprinting protocol^25^), as well as the ratio of the amount of communication in the original CQF^29^ to Q (*γ*_Q_). As shown in Table 1, for all the tested input sizes, *γ*_C_ and *γ*_Q_ are always larger than one, indicating that our WDM–CQF protocol not only requires less communication than the best-known classical protocol, but also beats the original CQF protocol. For large input size, our implementation even reduces more than half of the amount of communication in the CQF protocol.

**Table 1:** List of experimental parameters and experimental results.

Experimental details										
*n*	*M*	*μ*_A_	*μ*_B_	*C*_1,E_	*C*_1,D_	*C*_1,th_	*p*_error_	Q	*γ*_C_	*γ*_Q_
1.44 × 10^6^	1.0 × 10^6^	1282 ± 39	1479 ± 45	2.7 ± 0.1	34.3 ± 0.4	15	(2.7 ± 0.8) × 10^−5^	37321 ± 998	1.03 ± 0.03	1.26 ± 0.03
2.16 × 10^6^	1.5 × 10^6^	1425 ± 11	1644 ± 13	3.0 ± 0.2	38.4 ± 0.4	16	(1.1 ± 0.1) × 10^−5^	43792 ± 307	1.10 ± 0.01	1.22 ± 0.01
3.60 × 10^7^	2.5 × 10^7^	2724 ± 76	3143 ± 88	25 ± 2	96 ± 2	57	(3.2 ± 1.7) × 10^−6^	100176 ± 2567	1.92 ± 0.05	1.64 ± 0.04
7.19 × 10^7^	5.0 × 10^7^	3150 ± 105	3635 ± 121	53 ± 7	130 ± 10	87	(1.1 ± 0.6) × 10^−5^	121232 ± 3690	2.24 ± 0.07	1.90 ± 0.06
1.44 × 10^8^	1.0 × 10^8^	4050 ± 256	4673 ± 296	95 ± 4	202 ± 23	145	(1.6 ± 1.0) × 10^−5^	161422 ± 9406	2.4 ± 0.1	2.0 ± 0.1
3.60 × 10^8^	2.5 × 10^8^	6051 ± 469	6982 ± 541	251 ± 14	395 ± 24	309	(1.2 ± 0.9) × 10^−5^	250871 ± 17973	2.4 ± 0.2	2.0 ± 0.1
1.08 × 10^9^	7.5 × 10^8^	9722 ± 642	11218 ± 741	729 ± 41	958 ± 43	815	(9.4 ± 5.5) × 10^−6^	423574 ± 14812	2.5 ± 0.1	2.15 ± 0.08

*n*: size of the input string, *M* : total number of wavelength-composite pulses sent from Alice/Bob to Charlie. *μ*A and *μ*_B_ : total number of photons that are sent to Charlie by Alice and Bob respectively, *C*_1,E_ : total counts recorded by detector D_1_ when Alice and Bob have same inputs, *C*_1,D_ : total counts recorded by detector D_1_ when Alice and Bob have different inputs, *C*_1,th_ : threshold value of the total counts at detector D_1_, *P*_error_ : error probability, Q : amount of information communicated in our experiment. It is calculated as the equivalent number of qubits. *γ*_C_ : ratio of the amount of communication in the best-known classical fingerprinting protocol^25^ to Q ($32\sqrt{n}/Q$); *γ*_Q_ : ratio of the amount of communication in the original coherent fingerprinting protocol^29^ to Q.

**Fig. 5:**
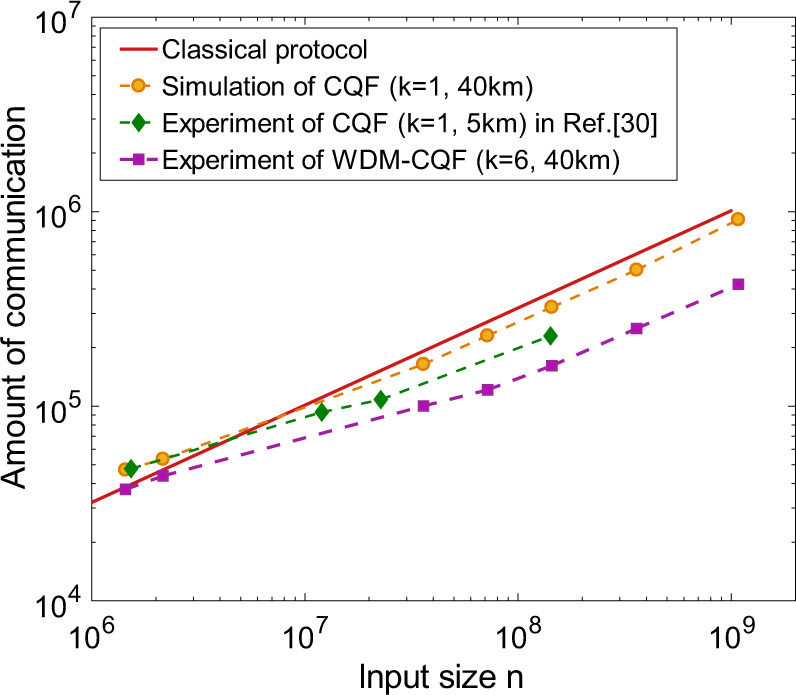
Log–log plot of the amount of communication between Alice/Bob and Charlie in different fingerprinting protocols, as a function of input size *n*. The solid red curve represents the best-known classical fingerprinting protocol^25^. The purple squares are the amount of communication in our demonstration of coherent quantum fingerprinting (CQF) with wavelength-division multiplexing (WDM). Six-wavelength channels are used. Except for the 6.9 km DCF, the overall distance between Alice and Bob is about 40 km. The orange circles correspond to the amount of communication in the original CQF system (*k* = 1) under the same experimental parameters. It is clear that less information is communicated in our experiment than that in both the classical fingerprinting and the original CQF protocol. We also plot out the amount of communication in another CQF experiment with a single-wavelength channel^30^ (green diamonds) for further comparison. Ref. ^30^ uses the same single-photon detectors as ours, but has a much shorter distance (only about 5 km). As shown, our WDM–CQF system outperforms the original CQF system.

The experimental results are also illustrated in Fig. 5, which is a log–log plot of the amount of communication in different fingerprinting protocols as a function of input size *n*. The solid red line represents the amount of communication required in the best-known classical fingerprinting protocol^25^. Our experimental results are represented by purple squares. The orange circles correspond to the simulation of the original CQF system with the same experimental parameters. Figure 5 clearly shows that our WDM–CQF system can beat the best-known classical fingerprinting protocol. More importantly, even with only six-wavelength channels, out system still significantly reduces the amount of communication in the original CQF protocol. For input size 1.44 × 10^6^ and 2.16 × 10^6^, the amount of communication in the CQF system with a single wavelength is even higher than the classical system. In our experiment, the amount of communication is always less than the best-known classical protocol. Especially for large input size, the advantage of using WDM is remarkable. For further comparison, we plot the experimental results reported in ref. ^30^, which uses the same SPDs (ID220) to demonstrate the original CQF protocol with a single-wavelength channel. Note that the total distance implemented in ref. ^30^ is only about 5 km, which is much shorter than the 40 km total distance in our implementation. Yet, the amount of information communicated in ref. ^30^ is much higher than our experimental results. This comparison further validates the fact that by applying WDM and using simultaneous detection, one can remarkably improve the performance of the original CQF protocol and make the system more robust to experimental imperfections (such as dark counts and channel losses).

## Discussion

Ideally, through applying six-wavelength channels, the communication time can also be decreased by a factor of six if all the wavelength components in each pulse are modulated simultaneously. But in our demonstration, we utilize the inherent chromatic dispersion of single-mode fibers to simplify the phase-modulation process. Considering that the six-wavelength components are phase-modulated one by one on Alice’s and Bob’s stations, our implementation does not strictly shorten the communication time. As a proof-of-concept demonstration, our experiment mainly proves that applying WDM to the CQF system can significantly reduce the amount of communication. To strictly show that our WDM–CQF protocol can also reduce the communication time in experiment, one can simply change the phase-modulation process to enable Alice and Bob to phase-modulate the wavelength components simultaneously. This can be done by replacing the use of temporal dispersion for phase modulation in our demonstration by the use of spatial dispersion.

The main limitation of our experimental implementation is the tolerable wavelength channels of our system. In order to avoid the pulse collision during the phase-modulation process, the span of different wavelength components on Alice’s and Bob’s stations should be at most half of the pulse-repetition period, that is, 10 ns in our system. Given the 800 ps modulation time for each channel and a channel spacing of 2.4 nm, at most 12-wavelength channels with a bandwidth around 26 nm can be applied to our system. One can change the corresponding experimental parameters (such as repetition rate, modulation window, and *δλ*) to increase the tolerable channel numbers. More importantly, through using spatial dispersion to replace temporal dispersion, the above limitation can be removed. When the number of wavelength channels is increased, the current polarization-alignment method may not work due to the increased bandwidth. In this case, one can use a polarizer at the end of a long-fiber spool to enforce the same polarization on different wavelength components. Since different wavelength components would undergo different attenuations by the polarizer, the signal intensities should be well adjusted to guarantee the same arrival intensities at Charlie’s station for different wavelength components. In our implementation, the intensity (*μ*/*m*) of each wavelength component is very low and channel spacing is not too narrow. Therefore, we ignore the possible cross talk^43,44^ between the adjacent channels and assume that the interference of pluses in each wavelength channel is independent. Further study about the cross-talk effect may be necessary if ultra-dense WDM (with a very small channel spacing *δλ*) is used.

In summary, we propose a variant of coherent-state-based quantum fingerprinting protocol with the use of WDM and simultaneous detection. We show that by using WDM, our proposed protocol can reduce the communication time of the original CQF protocol. More importantly, because of the simultaneous detection of many bits of information, the required amount of communication is significantly reduced in our WDM–CQF protocol. For an overall distance of as long as 40 km, our protocol with 100-wavelength channels can still beat the limit of classical fingerprinting without using SNSPDs. We also show that compared with the original CQF protocol, our WDM–CQF protocol can surpass the best-known classical fingerprinting protocol over a much longer distance. We have performed a proof-of-concept experimental demonstration of the WDM–CQF protocol with six-wavelength channels. The experimental results clearly show that the WDM–CQF scheme significantly outperforms both the classical and coherent fingerprinting protocols. Our practical and economical demonstration of quantum fingerprinting further validates the superiority of quantum communication complexity over its classical counterpart and shows the feasibility of real applications.

We remark that we propose to use WDM to improve the performance of the CQF protocol in this work. But WDM is not the only way. The key feature of our method is to take the advantage of detecting many bits of information simultaneously. One can use other types of multiplexing techniques, such as TDM, or even use a combination of various multiplexing schemes. Note that WDM would not help classical fingerprinting protocol reduce the amount of communication. This is because, in the classical fingerprinting protocol, no matter how many wavelength channels are used, at most one bit of information can be processed with a single pair of detectors. Moreover, in the classical fingerprinting scheme, each classical bit is often sent with many photons. While in our WDM–CQF protocol, many fewer photons are sent from the users to the central node. So, there is a huge saving in terms of the energy cost of communication too. It would be interesting to expand our method to other quantum communication protocols. In fact, in the coherent quantum fingerprinting system, the measurement on Charlie’s side is equivalent to a swap test, which has been applied in many other quantum communication protocols, such as quantum digital signature^45^. Our study introduces a promising method of using WDM to do such a test and shows the feasibility of applying WDM to other protocols.

Last but not least, in our implementation (and also in ref. ^30,31^), a two-way quantum communication system is used to ensure that Alice and Bob have the matched global phase. In this case, Alice’s and Bob’s station are actually physically connected. To remove this connection and to enable Alice and Bob independently prepare their fingerprints, one could also employ the method in ref. ^46^, where quantum fingerprinting based on higher-order interference is proposed and phase reference is not needed. An interesting question for future study could be whether we can still apply WDM to this method to further improve the communication efficiency. It would also be interesting to explore the possibility of using other degrees of freedom to increase the quantum channel capacity and make quantum communication more efficient.

## Methods

### Charlie’s decision mechanism

For Charlie to determine whether the inputs of Alice and Bob are equal or not, we adopt the method introduced in ref. ^30^ where a threshold value *C*_1,th_ is needed. When the total counts detected at Charlie’s detector *D*_1_ are smaller than the threshold *C*_1,th_, Charlie announces that the inputs *x* and *y* are the same. Otherwise, Charlie announces that the inputs are different. The choice of threshold *C*_1,th_ is dependent on the input size *n* and the total average photon number *μ*. The details of this decision mechanism are described as follows.

In each detection window, the probabilities for *D*_1_ obtaining a click for the equal inputs (*P*_E_) and 1-bit different inputs (*P*_D_) are given by Eqs. (6) and (7). In these two equations, *M* is the total number of pulses sent from Alice/Bob to Charlie and equals to

11 $$M=\frac{n/c}{k}=\frac{m}{k}.$$

As mentioned before, since each detection event is independent, the distributions of the total counts registered at D_1_ f or the equal and different input cases can be modeled as the binomial distributions B(*M*, *P*_E_) and B(*M*, *P*_D_), respectively. Moreover, in each detection window, there are *k* pairs of coherent states at different wavelengths interfering simultaneously. The distributions of the total counts at D_1_ depend on the total number of pulses *M* sent to Charlie and the total mean photon number *μ*. Moreover, for the same *M* and *μ*, the distributions of the total counts at D_1_ for the equal and different input cases are different, leading to different expectation values

12 $$\begin{array}{l}{\lambda }_{{{{\rm{E}}}}}=M\times {P}_{{{{\rm{E}}}}}\\ {\lambda }_{{{{\rm{D}}}}}=M\times {P}_{{{{\rm{D}}}}}.\end{array}$$

In the coherent fingerprinting scheme, the size of the inputs of interest is very large (*n* > 10^5^) and the detection probabilities *P*_E_ and *P*_D_ are always as small as the dark-count probability. Therefore, the above binomial distributions in this case are well described by the Poisson distributions Poi(*λ*_E_) and Poi(*λ*_D_).

**Fig. 6:**
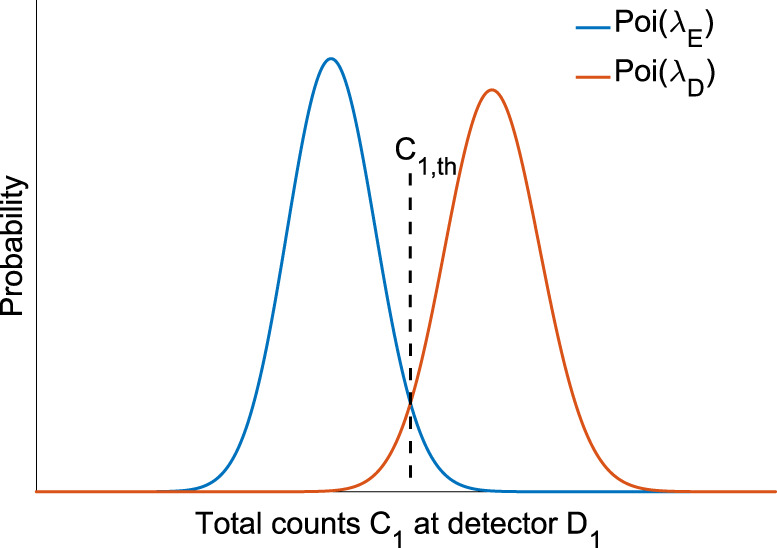
Probability distribution of the total counts at detector D_1_ for the equal-input case (blue curve) and different-input case (red curve). In this figure, the total number of pulses *M* is 5 × 10^9^. *δ* and *ν* in Eq. (6) and Eq. (7) are 0.22 and 97%, respectively. The detector’s dark-count rate is 100 Hz and *η* = 0.25 is considered as the detector efficiency (25%). The detection window is 500 ps. The average photon number *μ* is 1000. Note that this figure just shows an example of the probability distributions. Hence, the value of *μ* is not necessarily optimal and the corresponding error probability might be large.

Figure 6 shows an example of the distributions of the total counts at D_1_ for both the same-input case (blue curve) and different-input (red curve) cases. As indicated, the probability distributions for the two cases are away from each other. For most of the time, the total counts for different-input cases *C*_1,D_ are larger than the counts for the equal-input cases *C*_1,E_. Therefore, Charlie could choose a threshold total count *C*_1,th_ and compare *C*_1,th_ with the detected photon counts at D_1_. If the number of the detected counts is smaller (larger) than *C*_1,th_, Charlie concludes that Alice and Bob have the same (different) inputs. The errors exist when *C*_1,E_ is actually larger than the threshold, or *C*_1,D_ is smaller than the threshold. The error probability for Charlie’s decision is indicated by Eq. (8). As long as *P*_error_ is smaller than the tolerable error probability *ϵ*, Charlie’s conclusion is acceptable.

### Upper bound of error probability

In this section, an upper bound of Charlie’s error probability is discussed. For Poisson distributions, the Chernoff bound provides the upper bounds of probabilities P_r_(*C*_1,E_ > *C*_1,th_) and P_r_(*C*_1,D_ < *C*_1,th_) in Eq. (8) as

13 $${{{{\rm{P}}}}}_{{{{\rm{r}}}}}({C}_{1,{{{\rm{E}}}}}\, > \;{C}_{1,{{{\rm{th}}}}}) \, < \,\frac{{e}^{-{\lambda }_{{{{\rm{E}}}}}}{(e{\lambda }_{{{{\rm{E}}}}})}^{{C}_{1,{{{\rm{th}}}}}}}{{{C}_{1,{{{\rm{th}}}}}}^{{C}_{1,{{{\rm{th}}}}}}}\\ {{{{\rm{P}}}}}_{{{{\rm{r}}}}}({C}_{1,{{{\rm{D}}}}}\, < \,{C}_{1,{{{\rm{th}}}}}) \, < \,\frac{{e}^{-{\lambda }_{{{{\rm{D}}}}}}{(e{\lambda }_{{{{\rm{D}}}}})}^{{C}_{1,{{{\rm{th}}}}}}}{{{C}_{1,{{{\rm{th}}}}}}^{{C}_{1,{{{\rm{th}}}}}}},$$

as long as the threshold *C*_1,th_ is chosen to satisfy

14 $${\lambda }_{{{{\rm{E}}}}}\, < \,{C}_{1,{{{\rm{th}}}}}\, < \,{\lambda }_{{{{\rm{D}}}}}.$$

Moreover, if threshold *C*_1,th_ is the cross point of the two distributions Poi(*λ*_E_) and Poi(*λ*_D_), i.e.,

15 $${{{\rm{Poi}}}}({C}_{1,{{{\rm{th}}}}};{\lambda }_{{{{\rm{E}}}}})={{{\rm{Poi}}}}({C}_{1,{{{\rm{th}}}}};{\lambda }_{{{{\rm{D}}}}}),$$

then the upper bounds for the error probabilities P_r_(*C*_1,E_ > *C*_1,th_) and P_r_(*C*_1,D_ < *C*_1,th_) are the same. In this case,

16 $${C}_{1,{{{\rm{th}}}}}=\frac{{\lambda }_{{{{\rm{E}}}}}-{\lambda }_{{{{\rm{D}}}}}}{{{{{\rm{log}}}}}_{e}({\lambda }_{{{{\rm{E}}}}}/{\lambda }_{{{{\rm{D}}}}})}$$

and the upper bound for Charlie’s error probability is

17 $${P}_{{{{\rm{error}}}}}\, < \,{P}_{{{{\rm{upper}}}}}=\frac{{e}^{-{\lambda }_{{{{\rm{E}}}}}}{(e{\lambda }_{{{{\rm{E}}}}})}^{{C}_{1,{{{\rm{th}}}}}}}{{{C}_{1,{{{\rm{th}}}}}}^{{C}_{1,{{{\rm{th}}}}}}}.$$

### Optimization of *μ*

**Fig. 7:**
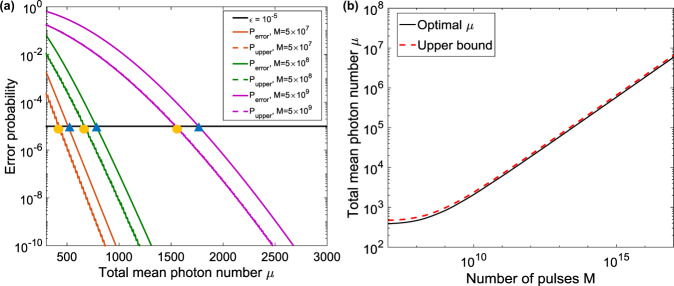
Optimization of averge photon number *μ*. **a** Log–log plot of error probability as a function of the total average photon number *μ*. Three different values of *M* (total number of pulses) are tested, that are 5 × 10^7^, 5 × 10^8^, and 5 × 10^9^. The solid curves indicate the error probability calculated based on Eq. (8) and Eq. (16). The yellow circles are the optimal *μ* chosen for different sizes of *M*. The dash curves are the upper bounds *P*_upper_ in Eq. (17) for different sizes of *M*. The blue triangles are the values of *μ* that satisfy Eq. (18). **b** Log–log plot of the total mean photon number *μ* as a function of the number of pulses *M* sent from Alice/Bob to Charlie. The black solid curve is the optimal *μ* searched for each *M*. The red dash curve is the upper bound of *μ* calculated from Eq. (18). In this simulation, an ECC with *c* = 0.2398 and *δ* = 0.22 is used. The interference visibility *ν* is assumed to be 97%. The detector’s dark-count rate is 100 Hz and *η* = 0.25 is considered as the detector efficiency (25%). The detection window is 500 ps.

As indicated by the above equations, the error probability depends on the total number of pulses *M* and the total mean photon number *μ*. If *M* and *μ* are known, one can use Eq. (8) to search an optimal threshold *C*_1,th_ (between *λ*_E_ and *λ*_D_) which gives the minimum error probability. As shown in Eq. (11), for a given system (ECC and *k* are fixed), *M* is only determined by the input size *n*. Now, the question is how to determine the average photon number *μ* for each value of *M*, as well as its corresponding optimal threshold *C*_1,th_. On one hand, *μ* should be large enough such that the detection probabilities (*P*_E_ and *P*_D_) are not dominated by *P*_dark_ and the error probability is below *ϵ*. On the other hand, since the amount of communication required is $Q=O(\mu {{{{\rm{log}}}}}_{2}n)$, *μ* should be as small as possible. Therefore, there is a trade-off between *P*_error_ and the minimum amount of communication *Q*. In our work, *C*_1,th_ is given by Eq. (16), which is a function of *M* and *μ*. Then for each value of *M*, the optimization of *μ* can be done by searching the minimum value of *μ* and the corresponding *C*_1,th_, which satisfies the error-probability condition, i.e., *P*_error_ < *ϵ*. As shown in Fig. 7a, the solid curves are the error probability *P*_error_ given in Eq. (8) as a function of the total number of photons *μ* for three different values of *M* (5 × 10^7^, 5 × 10^8^, and 5 × 10^9^). The optimal *μ* for different *M* is indicated by the yellow circle, the corresponding *P*_error_ of which is just below the tolerable error probability *ϵ* = 10^−5^ (black solid line).

Or, more simply, we do not have to search the optimal *μ* one by one. We can fix the upper bound of *P*_error_ to be equal to *ϵ*, i.e.,

18 $${P}_{{{{\rm{upper}}}}}=\frac{{e}^{-{\lambda }_{{{{\rm{E}}}}}}{(e{\lambda }_{{{{\rm{E}}}}})}^{{C}_{1,{{{\rm{th}}}}}}}{{{C}_{1,{{{\rm{th}}}}}}^{{C}_{1,{{{\rm{th}}}}}}}=\epsilon =1{0}^{-5}.$$

Then for each given *M*, one can directly calculate *μ* from the above equation. In this case, *P*_error_ can be always smaller than *ϵ*. In Fig. 7a, the dash curves are the upper bounds of *P*_error_ as a function of *μ* for different sizes of *M*. The calculated *μ* based on Eq. (18) for different *M* is indicated by the blue triangle. As shown in Fig. 7a, this calculated *μ* is larger than the optimal *μ*. For a small size of *M*, as in our experiment, searching the optimal *μ* can be done very quickly. For a very large size of *M*, searching optimal *μ* might be time-consuming, while directly calculating *μ* is very straightforward. Note that this calculated *μ* is actually the upper bound, as indicated in Fig. 7b. The black solid curve is the optimal *μ* as a function of *M* and the red dash curve is the upper bound of *μ* calculated based on Eq. (18). Since $Q=O(\mu {{{{\rm{log}}}}}_{2}n)$, for a given *n*, this upper bound of *μ* also gives the upper bound of the amount of communication in our WDM–CQF protocol. We remark that this upper bound might not be precise but fair enough as long as the Poisson distribution approximation used in “section A” is valid. The strict proof of this conclusion is out of the scope our paper.

### Validity of simultaneous detection of *k* pairs of wavelength components

**Fig. 8:**
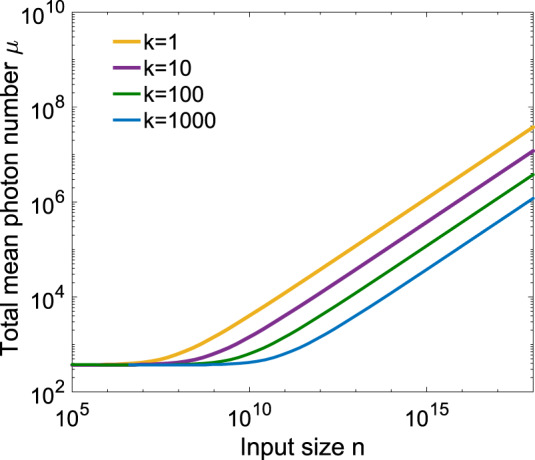
Log–log plot of the total average photon number *μ* required by the WDM–CQF protocol with different wavelength channels as a function of the input size *n*. When *k* = 1, the scheme becomes to the original CQF scheme. In this simulation, an ECC with *c* = 0.2398 and *δ* = 0.22 is used. The interference visibility *ν* is assumed to be 97%. The detector’s dark-count rate is 100 Hz and *η* = 0.25 is considered as the detector efficiency (25%). The detection window is 500 ps.

The advantage of transmitting less amount of information in our WDM–CQF protocol benefits from the shared detecting system for the *k* pair of wavelength components. As shown in Fig. 7b, the total average photon number *μ* is a function of the total number of pulses *M* sent out by Alice and Bob. In other words, as long as *M* is fixed, the average photon number in each wavelength-composite pulse is fixed, no matter how many wavelength components (*k*) it contains. For a fixed input size *n*, if more wavelength channels are used, the number of pulses sent from Alice/Bob to Charlie is reduced (*M* = *n*/(*ck*)). Consequently, less *μ* is required and the total amount of communication is reduced. Figure 8 shows the total average photon number *μ* required as a function of input size *n* for different values of *k*. It is clear that for large-input size, the more wavelength channels are applied, the smaller value of mean photon number is required.

In Eqs. (6) and (7), the *k* pair of wavelength components interferes simultaneously and are detected by a single pair of SPDs. As mentioned before, we assume that only the states in the same-wavelength channel would interfere with each other. In fact, in the coherent quantum fingerprinting protocol, to minimize the amount of communication, *μ* is always chosen to be so small that most of the pulses arriving at Charlie’s station are vacuum. At Charlie’s station, before the interference, the probabilities of each wavelength component being vacuum or having photons are

19 $${P}_{{{{\rm{vac}}}}}={e}^{-\frac{\mu \eta }{m}}$$

and (1 − *P*_vac_), respectively. For each pair of interfering pulses sent out by Alice and Bob, there are in total 2*k* components. Then, the probability that, more than one component, either from Alice or Bob, carries photons when arriving at Charlie’s station is given by

20 $$P=1-{{P}_{{{{\rm{vca}}}}}}^{2k}-2k\times (1-{P}_{{{{\rm{vac}}}}})\times {{P}_{{{{\rm{vac}}}}}}^{2k-1}.$$

**Fig. 9:**
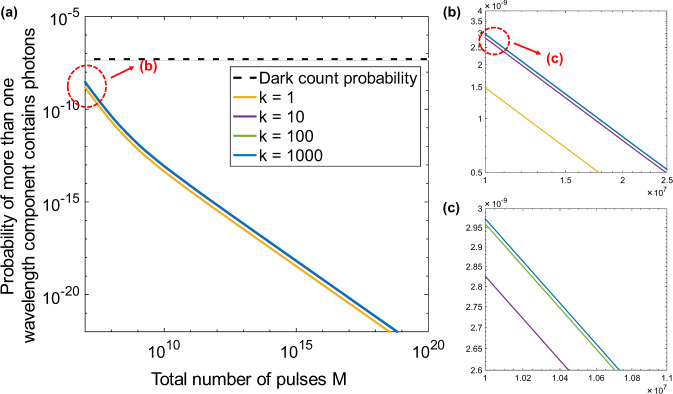
Probability of the case that, for each pair of pulses arriving at Charlie’s station, more than one-wavelength component contains photons as a function of *M*. Probabilities for different numbers of wavelength channels (k∈{1, 10, 100, 1000}). **a** Black dash line is the dark-count probability (5 × 10^−8^) considered in our simulation. **b** An enlarged figure of the red circle area in (a). (**c**): An enlarged figure of the red circle area in (b). In this simulation, an ECC with *c* = 0.2398 and *δ* = 0.22 is used. The interference visibility *ν* is assumed to be 97%. The detector’s dark-count rate is 100 Hz and *η* = 0.25 is considered as the detector efficiency (25%). The detection window is 500 ps.

In Fig. 9, we plot out the probability *P* as a function of the total number of pulses *M*. As shown in Fig. 9, for different values of *k*, this probability is always few orders of magnitude smaller than the dark count probability, which is around 5 × 10^−8^ in our simulation, especially for large *M*. As indicated in the enlarged Fig. 9b and Fig. 9c, when *k* increases from *k* = 1 to *k* = 1000, the increase of this probability is very small. That is to say, even for a value of *k* as large as 1000, we could ignore the case that more than one-wavelength component carries photons when each pair of pulses arrive at Charlie’s beam splitter. In this case, even there are *k* pairs of wavelength components that interfere simultaneously in each detection window, the multiwavelength contributions to the detection event are negligible. The detected clicks mainly come from the photons in one-wavelength component as well as the dark counts. Moreover, the information about which wavelength component has a photon is irrelevant, since Charlie’s decision is only determined by the total number of counts at detector D_1_. Therefore, in our scheme, demultiplexing is not needed on Charlie’s station and one pair of SPDs is adequate.

We remark that when *M* is relatively small (smaller than 10^6^), the above discussion would not be valid anymore, since the probability of the interfering pulses having more than one non-empty wavelength component would be too large to be ignored. Therefore, for the WDM–CQF system with different wavelength channels, the smallest size of input of interest is different. To benefit from applying a large number of wavelength channels, the input size should also be large.

## Supplementary information

Peer Review File

## Data Availability

The data generated during the study are available from the corresponding author upon reasonable request.
