# Supplementary material for: Efficient experimental quantum fingerprinting with channel multiplexing and simultaneous detection
Source: Nat Commun. 2021 Jul 22;12:4464. doi: 10.1038/s41467-021-24745-x (PMC8298536; doi:10.1038/s41467-021-24745-x)
Supplement: Supplementary file 1 — Peer Review File [file 41467_2021_24745_MOESM1_ESM.pdf]

Reviewers' Comments:

Reviewer #1:
Remarks to the Author:
The submission titled 'Efficient experimental quantum fingerprinting with wavelength division multiplexing' proposes the use of wavelength based multiplexing approach to solve the Equality problem with quantum fingeprints. Quantum fingerprinting is a well studied subject in quantum communication complexity which aims to achieve the task of Equality with exponenially less amount of communication compared to any classical protocol. The authors have tried to improve upon the existing coherent state fingerprinting protocol by the use of multiplexing.

In my opinion, it is interesting to see that this method further reduces on the amount of transmitted information cost, but at the cost adding substantially many lasers and keeping track of the increased caliberation challenges introduced by multiplexing. Even though the authors have proposed the proof of principle experiment with 6 different wavelengths, it is clearly not a scalable idea. As shown in the simulation plot of figure 2, to beat the classical lower bounds for a distance of 40km, even 1000 different wavelengths (as many different lasers in the setup) is not enough for SPD detectors of 10^-6 dark count and 20% efficieny. This means that for any reseasonable amount of distance, one comes back to same issue of adding SNSPD detectors. Even though this proposed protocol would perform better than the original coherent state coherent fingerprinting protocol, this comes at the cost of a huge amount of resources.

Another comparison that seems to be missing is that when the coherent state protocol can improve upon the resources by multiplexing, then so can the classical protocol. Hence, the fingerprinting protocol could have been formalised better where the authors could say that the protocol limits the processing of atmost one bit or one photon at a time. This would have motivated the idea that mulitplexing of the classical protocol would not help since Charlie is only processing one bit at a time.

In addition to this, the protocol has the same issue as the original quantum fingerprinting protocol, i.e. the use of sagnac loop. This means that Alice's pulses pass by Bob and Bob's by Alice. For the hope of any real world implementation of this protocol, this loop would not make sense because of privacy issues. Had the authors addressed this, it would been a substantially new and strong result.

In conclusion, the results of the paper is interesting but not strong enough to address the existing problems faced in the quantum fingerprinting protocol.

Reviewer #2:
Remarks to the Author:
In this paper, the authors present a new experimental approach to perform a quantum fingerprinting which a minimum of communication. This approach is a significant upgrade of the coherent quantum fingerprinting. Instead of using one wavelength to transfer the fingerprint, the authors propose to multiplex k different wavelength. To reduce the amount required communication the measurement is made without demultiplexing the wavelength. Like this, the mean photon number for each pulse remain the same and by increasing the number of wavelengths the mean photon number per bit of fingerprint is reduced. This approach is new and promising.

The paper is very clear with a detailed introduction on the fingerprinting protocol. The first part of the manuscript details the principle of this new approach and presents the theoretical model. Simulation results present the advantage of wavelength multiplexing as a function of the number of channels. The second part presents an experimental realization based on a Sagnac loop to avoid the problem of phase stabilization. The realization implemented over a distance of 40 km proves the pertinence of the approach.

The quality of the article and the novelty of the approach justify a publication in nature

communication without modification.

Reviewer #3:
Remarks to the Author:
The manuscript introduces a new quantum fingerprinting protocol employing coherent states and wavelength multiplexing. A proof of concept experiment is performed using 6 WDM channels. A single detection system is used to perform measurements on photons of all wavelengths.

The paper is clearly written and I believe the results are publishable in Nature Communications, provided the following points are addressed:

1) On section II, where the manuscript reads "the communication time is proportional to the input size n/c", I believe the authors meant "to the input size n" (as m = n/c).

2a) In Eq. (5), the parameter \eta represents the optical channel transmittance; however, there are two channels, Alice-Charlie and Bob-Charlie. Are the authors assuming symmetric (equal) losses? Are the detection efficiencies of D0 and D1 included in \eta?

2b) In the same equation, \nu is defined as the interference visibility. In fact, \nu depends on the loss mismatch on Alice's and Bob's sides, which may arise e.g. from different insertion losses of the optical components, different fiber lengths, and so on. Moreover, \nu also depends on polarization mismatches, time-of-arrival mismatches and so forth. The authors should comment on these dependencies and how they affect the basic protocol - maybe this makes a stronger case for employing your Sagnac solution, as the scheme of Fig. 1 is more general.

3) The distances involved in Fig. 2 are the Alice-Bob / Alice-Charlie one-way distances? Or do they take into consideration the full round trip the photons must perform under the Sagnac configuration that was adopted in the experiment?

4) The Sagnac implementation of Fig. 3 certainly circumvents many issues that would arise in other interferometric setups, especially phase stabilization. However, the photons must travel a much longer distance. Can this additional loss be somehow compensated by increasing the value of \mu?

5) In Fig. 3 there is a single polarization controller followed by a polarizer after the MUX. How can you ensure that the coherent states after the attenuator will have the same \alpha as in Eq. (2)?

6) On Table I, what are the units (i.e. the corresponding time windows) of \mu_A and \mu_B? Is the amount of communication Q expressed in bits?

7) In the discussion, the manuscript reads "When the number of wavelength channels is increased, the current polarization alignment method may not work due to the increased bandwidth". Do the authors suggest any alternative polarization control solutions to this problem?

8) The authors should explain the sentence "Through applying 1000 wavelength channels, the new WDM-CQF scheme with practical experimental conditions can even beat the limit of classical fingerprinting without using SNSPDs". First of all, would it be feasible to employ 1000 WDM channels using off-the-shelf components? Second, the first order approximation for PMD effects would no longer hold; how could PMD be circumvented for 1000 channels (i.e. a much higher bandwidth) in a practical scenario?

9) On Fig. 7, the time window considered for the calculation of \mu should be provided (see point 5 above).

10) A thorough spelling check is necessary on the manuscript. For
example, there is a typo on the legend of Fig. 2: "didoes" instead of "diodes". Many other typos were found during my reading.

Reviewer #1 (Remarks to the Author):

The submission titled 'Efficient experimental quantum fingerprinting with wavelength division multiplexing' proposes the use of wavelength based multiplexing approach to solve the Equality problem with quantum fingerprints. Quantum fingerprinting is a well studied subject in quantum communication complexity which aims to achieve the task of Equality with exponentially less amount of communication compared to any classical protocol. The authors have tried to improve upon the existing coherent state fingerprinting protocol by the use of multiplexing.

We thank the reviewer for the summary of quantum fingerprinting and appreciate his/her pointing out the merit of our work. In the following, we will address the reviewer's questions and concerns one by one. We have also revised our manuscript accordingly.

In my opinion, it is interesting to see that this method further reduces on the amount of transmitted information cost, but at the cost adding substantially many lasers and keeping track of the increased calibration challenges introduced by multiplexing.

R1. We thank the reviewer for raising this concern. On the one hand, we would like to point out that laser diodes are low-cost components and multiplexing of large number of channels (~ 100) is routinely used in today's telecom systems. On the other hand, we agree with the reviewer that there exist challenges to multiplex thousands of wavelength channels. We address the reviewer's concern in two ways:

(1) We have performed more simulation to show that, using the best commercially available single-photon detectors (with lower dark counts than those used in our simulation) can reduce the required number of wavelength channels. With around 100 channels, our quantum fingerprinting scheme with wavelength division multiplexing (WDM) can beat the classical limit over 40 km. See more details in the reply to the next comment.

(2) To achieve ~100 simultaneous channels, it is currently feasible to use WDM with a laser source for each channel. In addition, there are other alternative methods:

    a. One alternative is to use a combination of broadband source and spectral filters to create the different wavelength channels. This can be

done by a diffraction grating followed by spatial light modulations (both available commercially) for encoding.

b. Another method is to use other multiplexing schemes to create simultaneous channels. Our key message here is that our quantum fingerprinting scheme can take advantage of detecting many channels simultaneously. WDM is only one way to do multiplexing. One can also use time-division multiplexing (TDM), i.e., use fast modulators to add more temporal channels within one detection window. One can use other multiplexing schemes as well. One can also use a combination of various multiplexing schemes, such as TDM + WDM, to reduce the number of wavelength channels if needed. In short, we believe there are many practical ways to achieve ~100 simultaneous channels.

The above discussion of the possible solutions to achieve large number of wavelength channels and to reduce the cost of our WDM fingerprinting scheme has been added to our revised manuscript. Please see page 4, column 1, paragraph 1, starting from line 16.

Even though the authors have proposed the proof of principle experiment with 6 different wavelengths, it is clearly not a scalable idea. As shown in the simulation plot of figure 2, to beat the classical lower bounds for a distance of 40km, even 1000 different wavelengths (as many different lasers in the setup) is not enough for SPD detectors of 10^-6 dark count and 20% efficiency.

R2. We thank the reviewer for raising this point. First, we would like to clarify that, the distance shown in the plot of figure 2 in our manuscript is the length of fibers that connect between one user (Alice or Bob) and the central node (Charlie). To be consistent with other references, we re-define the distance in our revised manuscript to be the total length of fibers that connect between Alice and Charlie and fiber that connect between Bob and Charlie. For simplicity, we call it the overall distance between Alice and Bob. Now, we would like to address the reviewer's concerns with the following points.

1) Though our experimental demonstration using 6 wavelength channels over 40 km could not beat the classical limit, we have shown that the multiplexing scheme requires much less communication than both the original coherent quantum fingerprinting (CQF) protocol and the best-known classical protocol. Moreover, we have demonstrated that by applying

WDM, our fingerprinting scheme can show the quantum advantage over a much longer distance compared with the original CQF protocol.

2) For an overall distance of 80 Km, with the parameters of the single photon detector (SPD) in our lab (20% detection efficiency and 1000Hz dark count), our WDM scheme can not beat the classical limit with 1000 wavelength channels. However, we would like to point out that the performance of our WDM scheme can be much improved with a pair of best available commercial SPDs. For example, ID230 from ID Quantique has a quantum efficiency up to 25% and a dark rate as low as 50 Hz. We have now performed another simulation with a quantum **efficiency of 25%** and **a dark count rate of 100 Hz**. The results are shown in Fig.1.

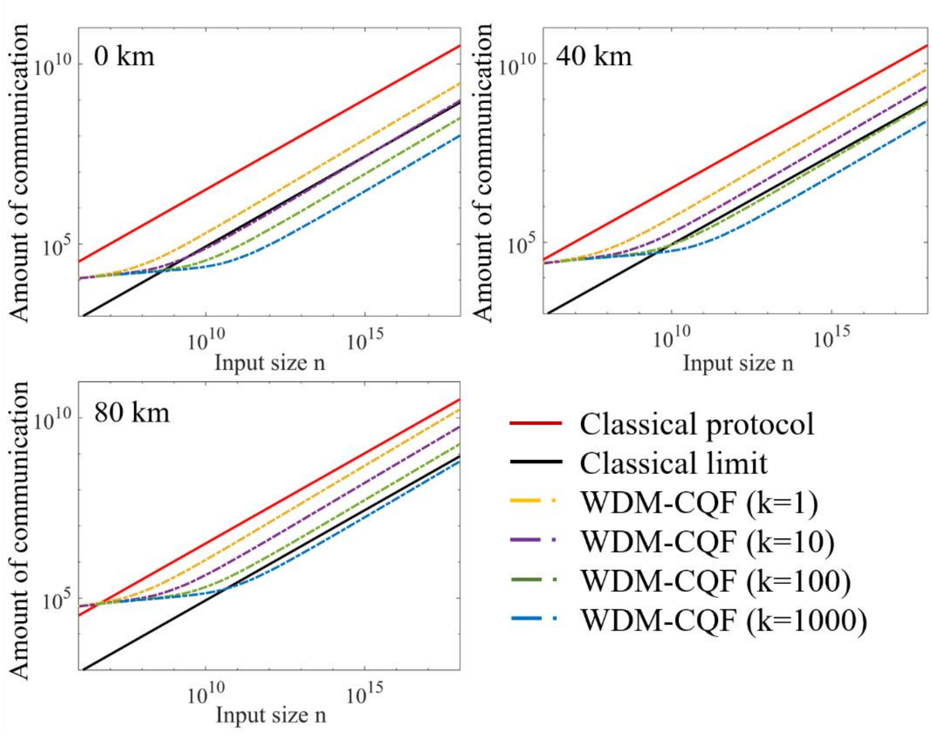

Figure 1: Amount of information communicated in different fingerprinting schemes.

a) As you can see, with the new parameters, our WDM scheme can beat the classical limit at 40 km with only 100 wavelength channels. As mentioned in our reply to the previous comment (R1.2), it is feasible to achieve ~100 wavelength channels with current technologies.

b) For an overall distance of 80 Km, our WDM scheme can also beat the classical limit with 1000 wavelength channels. We admit that using 1000 wavelength channels in our current set-up can be challenging, but this number can potentially be reduced by the methods mentioned in (R1.2b) of the reply to the previous comment.

3) We did another simulation where the overall distance between Alice and Bob is about 20 Km. It turned out that with ~30 wavelength channels, our WDM-CQF protocol can beat the classical limit without the use of superconducting nanowire SPD (SNSPD). The result is shown in Fig. 2 below. Note that in the most recent experimental demonstration of the original CQF protocol, the longest overall distance tested is also 20 km (Ref. [31]). The amount of communication in Ref. [31] is less than the classical limit, but with the use of SNSPD. We remark that laser diodes and modulators are commonly used in telecom systems and are low cost, compared to SNSPDs.

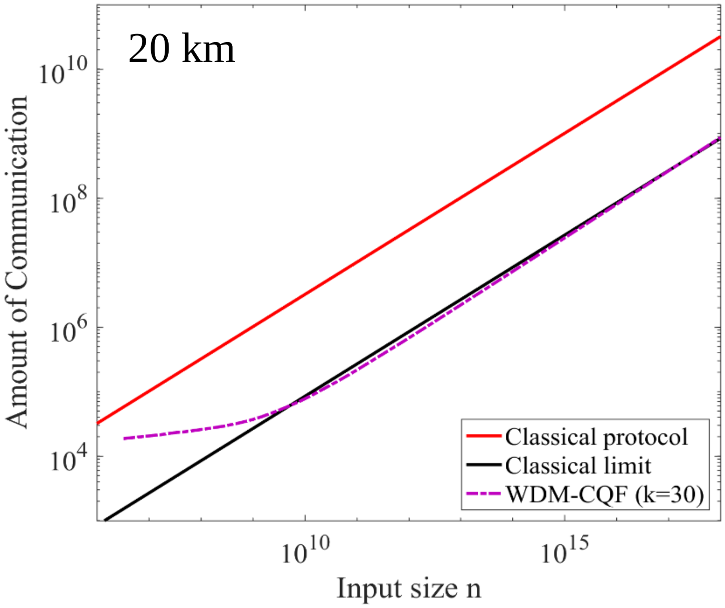

Figure 2: Amount of information communicated in the WDM-CQF scheme with 30 wavelength channels. The overall distance between Alice and Bob is 20 km.

In our revised manuscript, figure 2 has been replaced with the new simulation plot. The discussion of our simulated results has also been revised accordingly. Please see page 3, column 2, last paragraph.

This means that for any reasonable amount of distance, one comes back to same issue of adding SNSPD detectors. Even though this proposed protocol would perform better than the original coherent state coherent fingerprinting protocol, this comes at the cost of a huge amount of resources.

R3. We appreciate the reviewer's comment. As the reviewer has mentioned, by using WDM, our protocol performs much better than the original CQF protocol. That is to say, our WDM-CQF protocol can achieve quantum advantage over much

longer distance than the original CQF protocol. We remark that the increased cost of resources for applying WDM to the original CQF protocol mainly comes from the signal preparation and signal detection stages. But in our WDM scheme, due to the fact of the simultaneous detections of different wavelength components, only a single pair of detectors is needed. Therefore, no extra resources are added in the detection stage.

In the signal preparation stage, our scheme does require a large number laser diodes when many wavelength channels are needed. However, laser diodes are low cost (<$100 for large quantities), so the total cost of added laser diodes are low compared with SPDs and SNSPDs.

Alternatively, instead of using many laser diodes, other methods can be used, as mentioned in part (R1.2) of the reply to the previous comment.

Another comparison that seems to be missing is that when the coherent state protocol can improve upon the resources by multiplexing, then so can the classical protocol. Hence, the fingerprinting protocol could have been formalised better where the authors could say that the protocol limits the processing of at most one bit or one photon at a time. This would have motivated the idea that multiplexing of the classical protocol would not help since Charlie is only processing one bit at a time.

R4. We greatly appreciate this suggestion from the reviewer. This is a good point which we have added into the discussion of our manuscript.

We agree with the reviewer that multiplexing would not help classical fingerprinting protocol reduce the amount of communication. This is because in the classical protocol, no matter how many wavelength channels are used, at most one bit of information can be processed with a single pair of detectors.

But in the *quantum* fingerprinting protocol, multiplexing can enable processing of multiple bits of information at the same time. Moreover, in the classical fingerprinting scheme, each classical bit is often sent with many photons. While in our WDM-CQF protocol, many fewer photons are sent from the users to the central node. So, there is a huge saving in terms of the energy cost of communication too.

Please see this discussion in page 6, column 2, last paragraph.

In addition to this, the protocol has the same issue as the original quantum fingerprinting protocol, i.e. the use of sagnac loop. This means that Alice's pulses pass by Bob and Bob's by Alice. For the hope of any real world implementation of this protocol, this loop would not make sense because of privacy issues. Had the authors addressed this, it would been a substantially new and strong result.

R5. We thank the reviewer for this question.

First, our WDM-CQF protocol itself does not require the Sagnac loop configuration. Alice and Bob can prepare their stages separately and send the states to Charlie for measurement. The Sagnac loop is only used in our proof-of-principle experimental demonstration.

Second, in our experiment, there is no information transmitted between Alice and Bob. Inside the Sagnac loop, the clockwise traveling pulse first passes through Alice's station without any modulation done by Alice. That is to say, no information is carried by the pulse when it enters Bob's station. Then Bob encodes his information into the phase of the pulse and sends it back to Charlie. So does the counter-clockwise traveling pulse, no information from is encoded into the counter-clockwise traveling pulse even it passes through Bob's station. Only when it enters Alice's station, it is then phase modulated and forwarded back to Charlie's station. Therefore, there is no privacy issue.

Third, we would like to point out that, unlike quantum key distribution, the purpose of quantum fingerprinting is to reduce communication complexity, rather than to achieve privacy. Privacy and security issues are often considered to be outside the scope of the protocol.

We have revised our manuscript to make this point clearer for the readers. Please see page 4, column 2, paragraph 1, line 11-15.

In conclusion, the results of the paper is interesting but not strong enough to address the existing problems faced in the quantum fingerprinting protocol.

Once again, we sincerely thank the reviewer for her/his valuable suggestions, which helped us significantly strengthen our manuscript. We hope that with our reply, as well as with the revised manuscript, we have now properly addressed his/her concerns about our work, and he/she could recommend its publication in Nature Communication.

Reviewer #2 (Remarks to the Author):

In this paper, the authors present a new experimental approach to perform a quantum fingerprinting which a minimum of communication. This approach is a significant upgrade of the coherent quantum fingerprinting. Instead of using one wavelength to transfer the fingerprint, the authors propose to multiplex k different wavelength. To reduce the amount required communication the measurement is made without demultiplexing the wavelength. Like this, the mean photon number for each pulse remain the same and by increasing the number of wavelengths the mean photon number per bit of fingerprint is reduced. This approach is new and promising.

The paper is very clear with a detailed introduction on the fingerprinting protocol. The first part of the manuscript details the principle of this new approach and presents the theoretical model. Simulation results present the advantage of wavelength multiplexing as a function of the number of channels. The second part presents an experimental realization based on a Sagnac loop to avoid the problem of phase stabilization. The realization implemented over a distance of 40 km proves the pertinence of the approach.

The quality of the article and the novelty of the approach justify a publication in nature communication without modification.

We greatly appreciate the reviewer's approval of our manuscript. The reviewer has completely summarized our manuscript and pointed out the merits of our work. We thank the reviewer for his/her recommendation of our manuscript to the publication in Nature Communication.

Reviewer #3 (Remarks to the Author):

The manuscript introduces a new quantum fingerprinting protocol employing coherent states and wavelength multiplexing. A proof of concept experiment is performed using 6 WDM channels. A single detection system is used to perform measurements on photons of all wavelengths.
The paper is clearly written and I believe the results are publishable in Nature Communications, provided the following points are addressed:

We first thank the reviewer for recommending the publication of our paper in Nature Communication. The reviewer's comments and suggestions are very helpful. We would like to address these points one by one below.

1) On section II, where the manuscript reads "the communication time is proportional to the input size n/c", I believe the authors meant "to the input size n" (as m = n/c).

R1. We thank the reviewer for pointing out this confusion. Yes, the communication time is proportional to the input size n. We have corrected this sentence.

2a) In Eq. (5), the parameter \eta represents the optical channel transmittance; however, there are two channels, Alice-Charlie and Bob-Charlie. Are the authors assuming symmetric (equal) losses? Are the detection efficiencies of D0 and D1 included in \eta?

R2. a) We thank the reviewer for pointing out the unclear definition of η. Yes, both the channel loss and detectors' efficiency should be considered into η, i.e. $\eta = \eta_{trans} * \eta_{det}$. Here we consider the symmetric case where $\eta_A = \eta_B$. However, if the channel losses are different, then Alice and Bob simply send different signal intensities to compensate the channel asymmetry, that is to say $\mu_A * \eta_A = \mu_B * \eta_B$.

We have now revised the definition of η. Please see page 3, column 1, line 6-11 from the bottom.

2b) In the same equation, \nu is defined as the interference visibility. In fact, \nu depends on the loss mismatch on Alice's and Bob's sides, which may arise e.g. from different insertion losses of the optical components, different fiber lengths, and so on. Moreover, \nu also depends on polarization mismatches, time-of-arrival mismatches and so forth. The authors should comment on these dependencies and how they affect the basic protocol - maybe this makes a stronger case for employing your Sagnac solution, as the scheme of Fig. 1 is more general.

R2. b) We thank the reviewer for this good suggestion. We totally agree with the reviewer that a lot of factors would contribute to the imperfection of interference and decrease the interference visibility. Therefore, in a general set-up where Alice and Bob prepare their states separately, as shown in Fig. 1 in the manuscript, careful calibrations of total losses, phase stability and good polarization alignment are required.

However, as the reviewer suggested, all these requirements can be easily achieved by using a Sagnac interferometer, as shown in our experimental demonstration. Because of the common path for signals traveling inside the loop, The Sagnac loop automatically compensate the phase and polarization fluctuation as well as the losses mismatch.

We have now included this discussion in our revised manuscript. Please see page 3, column 1, line 12-18 from the bottom and page 4, column 1, line 6-11 in the first paragraph of subsection “Experimental set-up”

3) The distances involved in Fig. 2 are the Alice-Bob / Alice-Charlie one-way distances? Or do they take into consideration the full round trip the photons must perform under the Sagnac configuration that was adopted in the experiment?

R3. We thank the reviewer for pointing out this unclear definition. The distance shown in Fig. 2 in our manuscript is the one way distance between one user (Alice or Bob) and the central node (Charlie). To be consistent with other references, in our revised manuscript as well as in this reply letter, we re-define the distance to be the total length of fibers that connect between Alice and Charlie and fibers that connect between Bob and Charlie. For simplicity, we call it the overall distance between Alice and Bob. Note that in our experiment, the length of fibers that directly connecting between Alice and Bob should not be considered into the overall distance.

The definition of the distance can be found in page 3, column 2, line 4-7 in the last paragraph.

4) The Sagnac implementation of Fig. 3 certainly circumvents many issues that would arise in other interferometric setups, especially phase stabilization. However, the photons must travel a much longer distance. Can this additional loss be somehow compensated by increasing the value of \mu?

R4. We agree with the reviewer that the pulses would travel a longer distance because of this loop configuration. This could be compensated by Charlie who

could send a stronger pulse into the loop. We would like to clarify that the signal intensity μ, measured at the output of Alice's\Bob's station, would not be affected. This is because the signal state which carries the information from Alice\Bob would only travel through the fibers connecting between Alice\Bob and Charlie, not the whole loop.

5) In Fig. 3 there is a single polarization controller followed by a polarizer after the MUX. How can you ensure that the coherent states after the attenuator will have the same \alpha as in Eq. (2)?

R5. We appreciate this good question from the reviewer. Before we add attenuation on Charlie's station, we measure the power of each wavelength component at the output of the attenuator, by using a calibrated power-meter. The output power of each laser module is individually adjusted to make sure that after the attenuator, each wavelength component has the same intensity. Then we increase the attenuation to create weak coherent states. Here we assume that the attenuation of the attenuator is wavelength independent.

We have clarified this in the manuscript in page 4, column 1, last paragraph, line 1-3 from the bottom.

6) On Table I, what are the units (i.e. the corresponding time windows) of \mu_A and \mu_B? Is the amount of communication Q expressed in bits?

R6. We thank the reviewer for pointing out this unclear point in our manuscript. μ_A and μ_B in Table 1 represent the total number of photons that Alice and Bob send to Charlie during each test. The average photon number in each wavelength composite pulse would be μ/M. The detection window is the same as the pulse width, which is 500 ps. The unit for the amount of communication Q in the quantum fingerprinting protocol is qubit. We have now clarified theses units in our revised manuscript.

7) In the discussion, the manuscript reads "When the number of wavelength channels is increased, the current polarization alignment method may not work due to the increased bandwidth". Do the authors suggest any alternative polarization control solutions to this problem?

R7. We appreciate this good point raised by the reviewer. When the operation bandwidth is much larger, say tens of nanometers, then we can use a polarizer at the end of a long fiber spool to enforce the same the polarization on different

wavelength components. Since different wavelength components would undergo different attenuations by the polarizer, the signal intensities of different wavelength components should be well adjusted to guarantee that the arrival intensities at Charlie's station are the same.

We have included this discussion in our revised manuscript. Please see page 6, column 2, line 9-15.

8) The authors should explain the sentence "Through applying 1000 wavelength channels, the new WDM-CQF scheme with practical experimental conditions can even beat the limit of classical fingerprinting without using SNSPDs". First of all, would it be feasible to employ 1000 WDM channels using off-the-shelf components? Second, the first order approximation for PMD effects would no longer hold; how could PMD be circumvented for 1000 channels (i.e. a much higher bandwidth) in a practical scenario?

R8. We thank the reviewer for this helpful suggestion that we should address the challenges of applying 1000 wavelength channels. We agree with the reviewer that multiplexing 1000 wavelength channels might be infeasible with current technology. One solution to circumvent this larger number of wavelength channels is using a combination of wavelength division multiplexing (WDM) and time-division-multiplexing (TDM). For each wavelength channel, we can use a fast modulator to encode m (larger than 1) bits of information on m pulses within one detection window. In this case, only 1000/m wavelength channels are needed to beat the classical limit. Our key message here is that our quantum fingerprinting scheme can take advantage of detecting many channels simultaneously. WDM is only one way to do multiplexing. We can combine WDM with other type of multiplexing techniques to reduce the required number of wavelength channels.

We would also like to mention that in the simulation of Fig. 2 in our manuscript, we used the parameters of single photon detectors (SPD) in our lab (20 % quantum efficiency and 1000 Hz dark count rate). We have performed more simulations with parameters from the better commercially available SPDs (ID230 from ID Quantique, 25% quantum efficiency and 100 Hz dark count rate). We show that much less wavelength channels are required to beat classical limit. For instance, for an overall distance of 20 Km, our WDM fingerprinting protocol can beat the classical limit with only 30 wavelength channels, which is feasible to be implemented. The new simulation results are shown in Fig. 1 and Fig. 2 in our

reply to the first reviewer. We have also revised our manuscript to show the new simulation results (including replacing the simulation plots).

The above discussion has been added in our revised manuscript. Please see page 4, column 1, paragraph 1, starting from line 16.

9) On Fig. 7, the time window considered for the calculation of \mu should be provided (see point 5 above).

R9. We thank the reviewer again for this suggestion. In our new simulation, the detection window is always 500 ps, which matches the detection window used in our experiment. We have now indicated the time window clearly in each figure in our revised manuscript.

10) A thorough spelling check is necessary on the manuscript. For example, there is a typo on the legend of Fig. 2: "didoes" instead of "diodes". Many other typos were found during my reading.

R10. We appreciate the reviewer pointing out these typos. We have thoroughly checked our manuscript and corrected them.

Again, we sincerely thank the reviewer for his/her insightful suggestions and comments, which help us significantly improve our manuscript. We hope that our reply has addressed the concerns from the reviewer. we have also revised our manuscript in accordance with the reviewer’s comments. We hope that the reviewer will now find our paper suitable for publication.

Reviewers' Comments:

Reviewer #4:
Remarks to the Author:
In this paper the authors present the experimental implementation of a coherent state quantum fingerprinting protocol that includes multiplexing into multiple frequency channels and detection by a pair of single-photon detectors. Although the idea of using multiple channels for quantum fingerprinting has been previously introduced as a means to gain in communication time, here the authors don't show such an advantage due to experimental reasons but rather focus on a gain in transmitted information and also highlight the simplicity of using only two detectors to achieve this. I find the paper interesting and new results in practical quantum fingerprinting (in particular, over a significant distance like in this case) are welcome and timely, but I believe that the claim of an additional advantage in transmitted information the authors make is problematic. This reduces the potential impact of the paper, and hence I cannot recommend it for publication in Nature Communications.

My main issue regarding this claim is that the authors don't look in fact at how much transmitted information is sent from Alice and Bob but rather at how much is left after Bob applies his detection strategy, which essentially amounts to leaving out lots of information. In other worlds, what happens is that from the dimension of the Hilbert space of the message at Alice's we arrive at Bob, who projects this space into a much smaller one by his measurement strategy, where many frequency channels have been multiplexed in the same time slot and are detected altogether by the two detectors. So, this essentially depends on this 'suboptimal' Bob and the lower transmitted information is not an inherent property of the protocol. Hence, the results cannot be framed in my opinion as a gain in the amount of the transmitted information (or amount of communication).

I also found it disappointing that the authors have not shown an improvement in communication time with their experiment. This is in general an important impairment of coherent quantum fingerprinting protocols and it would be important for the authors to at least discuss how an improvement could be reached.

A less important remark is that I found it confusing that the simulations are provided for different detector characteristics than the ones used in the experiment. I understand that this was done to answer one of the reviewer's comments but the result lacks coherence. I would recommend to the authors to make this more consistent. They could also show what performance they would get with SNSPDs. The argument that such detectors are not required is not particularly compelling in this particular case, as only two detectors are necessary for the multiplexed scheme and so using such 'expensive' detectors is not a very big issue.

The authors should also justify more clearly why they have neglected multi-photon contributions in their analysis.

As one of the reviewers has also pointed out, the experimental setup based on a Sagnac loop lacks elegance with Alice and Bob being physically connected (as also acknowledged by the authors). It also leads to some unnatural (and possibly not entirely justified) choices, like excluding the DCF fiber from the total distance calculation, although its presence is integrant to the experimental implementation.

Reviewer #4 (Remarks to the Author):

In this paper the authors present the experimental implementation of a coherent state quantum fingerprinting protocol that includes multiplexing into multiple frequency channels and detection by a pair of single-photon detectors. Although the idea of using multiple channels for quantum fingerprinting has been previously introduced as a means to gain in communication time, here the authors don't show such an advantage due to experimental reasons but rather focus on a gain in transmitted information and also highlight the simplicity of using only two detectors to achieve this. I find the paper interesting and new results in practical quantum fingerprinting (in particular, over a significant distance like in this case) are welcome and timely, but I believe that the claim of an additional advantage in transmitted information the authors make is problematic. This reduces the potential impact of the paper, and hence I cannot recommend it for publication in Nature Communications.

We sincerely thank the reviewer for taking time to review our manuscript, and note that he/she thinks our work is "new" and "timely". We believe the reviewer's concern is due to some misunderstandings about our manuscript (which we will explain in detail below), and we hope that once this misunderstanding is cleared, our manuscript will be considered for publication in Nature Communications.

Regarding the main claim of an advantage in transmitted information and other issues raised by the reviewer, below is our detailed response.

1) My main issue regarding this claim is that the authors don't look in fact at how much transmitted information is sent from Alice and Bob but rather at how much is left after Bob applies his detection strategy, which essentially amounts to leaving out lots of information. In other worlds, what happens is that from the dimension of the Hilbert space of the message at Alice's we arrive at Bob, who projects this space into a much smaller one by his measurement strategy, where many frequency channels have been multiplexed in the same time slot and are detected altogether by the two detectors. So, this essentially depends on this 'suboptimal' Bob and the lower transmitted information is not an inherent property of the protocol. Hence, the results cannot be framed in my opinion as a gain in the amount of the transmitted information (or amount of communication).

   Firstly, in a quantum fingerprinting scheme, Alice and Bob are the users who send their fingerprints to the referee, Charlie, who performs measurement and announces the result of comparison. In the reviewer's comment, the referee is miswritten as Bob.

   Secondly, we would like to clearly state that, the amount of information calculated in our manuscript is in fact the amount of information sent from the users (Alice and Bob) to the referee (Charlie). We calculate this information [equation (3)] based on the definition given in the original coherent quantum fingerprinting (CQF) paper [29], and is exactly the same as in other experimental CQF demonstrations [30,31].

   In the original CQF [29], to compare a pair of input strings $x, y \in \{0,1\}^n$, the amount of transmitted information is $O(\mu log_2 m)$, where $m(=\frac{n}{constant})$ is the number of coherent

pulses sent by Alice or Bob to Charlie and μ is the total average photon number. In our method, even though Alice or Bob only sends $M(=\frac{m}{k})$ coherent pulses with each pulse containing k wavelength components, the amount of transmitted information is still calculated as $O(\mu log_2 m)$, not $O(\mu log_2(\frac{m}{k}))$. Note that this calculation only depends on the size n of the input strings and the total average photon number μ. It is independent of Charlie's measurements. We believe the reviewer misunderstood our calculation.

Thirdly, referring to reviewer's comment that "many frequency channels have been multiplexed in the same time slot and are detected altogether by the two detectors": the fact that Charlie's measurements do not require demultiplexing is a distinct ***feature and advantage*** of our new CQF protocol presented in this work. It is considerably simpler and more practical to implement.

The reason we can do without demultiplexing is because, in our CQF protocol the probability of more than one wavelength component in each wavelength-composite pulse containing photons is lower than the dark count probability. Our detailed calculation has shown that multi-wavelength contributions can be ignored. (Please find the detailed explanation in our reply to comment 4). More detailed analysis can be found on page 8, part D of the "Method" section in our manuscript).

Lastly, the reason we have greater gain of the amount of communication with our new protocol (compared to the original CQF protocol) is that our protocol requires a smaller μ compared with the original CQF protocol. As shown in equations (6) and (7), because of the simultaneous detection of k pairs of wavelength components, the signal detection probability relative to the error detection probability is increased. (More detailed discussion can be found on page 3, part A of the "Result" section in our manuscript.) Therefore, to compare the input strings with the same error probability, the required average photon number μ in our new protocol can be smaller than that in the original CQF protocol. Hence, the amount of information sent from Alice and Bob to Charlie $O(\mu log_2 m)$ in our protocol is less that that in the original CQF protocol.

We have revised our manuscript to clearly state that the calculation of the amount of transmitted information from Alice and Bob to Charlie in our protocol is the same as the calculation in Ref. [29]. Please see the revisions on page 2, column 2, the two lines above Eq. (3) and page 3, column 1, lines 1-3. We have also made our claim of the gain in the amount of communication clearer, showing that the reduced amount of communication is due to the decreased average photon number μ. Please see the revisions on page 3, column 2, paragraph 2.

2) I also found it disappointing that the authors have not shown an improvement in communication time with their experiment. This is in general an important impairment of coherent quantum fingerprinting protocols and it would be important for the authors to at least discuss how an improvement could be reached.

We agree improvement in communication time is desirable, and we have in fact pointed out in our manuscript (on page 2) that by applying WDM, our proposed protocol can reduce the communication time to $1/k$ times of its original value if all wavelength channels are modulated simultaneously. In our experimental demonstration, however, Alice/Bob has only one modulator each, so the channels are modulated in sequence. In other word, we trade-off modulation hardware with communication time. In addition, we did discuss about how to adapt our current set-up to show that the communication time can be reduced with our new protocol on page 6. At its simplest, one can use spatial dispersion rather than temporal dispersion during the modulation process of Alice and Bob to gain advantage of less communication time.

We have now revised our manuscript to make this discussion clearer. Please see the revision on page 6, column 2, paragraph 1.

3) A less important remark is that I found it confusing that the simulations are provided for different detector characteristics than the ones used in the experiment. I understand that this was done to answer one of the reviewer's comments but the result lacks coherence. I would recommend to the authors to make this more consistent. They could also show what performance they would get with SNSPDs. The argument that such detectors are not required is not particularly compelling in this particular case, as only two detectors are necessary for the multiplexed scheme and so using such 'expensive' detectors is not a very big issue.

We thank the reviewer for the suggestions. In our simulation, we use the parameter from the best available commercial single-photon avalanche photodiodes (SPAPDs) to show that our new coherent fingerprinting protocol can practically beat the classical limit without using superconducting nanowire SPDs (SNSPDs). Though a pair of SNSPDs might be affordable for the purpose of lab demonstrations, in the view of practical applications, the cheaper SPAPDs would be a better choice.

4) The authors should also justify more clearly why they have neglected multi-photon contributions in their analysis.

We thank the reviewer for this suggestion. Actually, we do have a detailed discussion about why multi-photon contributions are negligible in the "Method" section. Please see this discussion on page 8, part D of the 'Method' section in our manuscript.

Note that, by saying ignoring multi-photon contributions, we really mean that we can ignore the case where multiple wavelength components contain photons during each detection window and contribute to the detection event. Let k be the number of the wavelength channels. As shown in our analysis, for a value of k as large as 1000, the probability of more than one wavelength component in a pair of pulses (with 2k wavelength components) containing photons when arriving at Charlie's station is even lower than dark count probability. Therefore, in each detection window, the contributions to the detection event mainly come from the photons in one wavelength component as well as the dark counts. Moreover, the information of which wavelength component contributing to detection event is not needed. Hence, de-multiplexing can be removed in our method and k pairs of coherent

states at different wavelengths interfere simultaneously and are detected by a single pair of detectors.

To clarify our point, we have now replaced "multi-photon contributions" by "multi-wavelength contributions". We have also revised our manuscript to point out more clearly that we have fully calculated the multi-wavelength contributions and found them to be negligible. See page 3, column 2, paragraph 2.

5) As one of the reviewers has also pointed out, the experimental setup based on a Sagnac loop lacks elegance with Alice and Bob being physically connected (as also acknowledged by the authors). It also leads to some unnatural (and possibly not entirely justified) choices, like excluding the DCF fiber from the total distance calculation, although its presence is integrant to the experimental implementation.

We would like to point out that, 1) the signal traveling between Alice and Bob in our set up are unmodulated, i.e, that they do not contain information and therefore there is no information exchange directly between Alice and Bob; and 2) whether Sagnac loop is used or some other configuration is used [30][31], there always needs (unmodulated) signals traveling between Alice and Bob for phase reference or phase stabilization. In Ref. [30] where a plug and play QKD system is used, Referee Charlie first sends a reference (signal) pulse to Alice (Bob) who just lets the pulse pass through the station without any modulation. When the reference (signal) pulse arrives at Bob's (Alice's) station, it is then phase modulated by Bob (Alice) and is forwarded back to Referee's station for measurement. In Ref. [31], a so-called 'zero-area' Sagnac type interferometer is also used in the set-up. It works just like the Sagnac loop, where signals always first pass through one user's station without any modulation, then arrive at another user's station for phase modulation and finally go back to Referee's station for measurement. Because of the need for common phase reference between Alice and Bob, there will always be signals traveling between Alice and Bob directly, regardless of what configuration is used. So we do not agree with the reviewer that our Sagnac configuration "lacks elegance with Alice and Bob being physically connected".

As for the total distance calculation, we only consider the distances between Alice and Charlie and between Bob and Charlie in our experiment, so that the calculated total distance is consistent with the theorical model, and with other QF demonstrations. Note that if we had included the length of DCF fibers in our calculation, the total communication distance of our demonstration would have been even longer, which would not affect the validity of our experimental results at all, but actually would make our claim even stronger.

Lastly, in our manuscript, we have discussed the possible method to remove the fiber link between Alice and Bob for the implementation of quantum fingerprinting protocol. "To remove this connection and to enable Alice and Bob independently prepare their fingerprints, one could also employ the method in Ref. [48], where quantum fingerprinting based on higher order interference is proposed and phase reference is not needed." This discussion can be found on page 7, the last paragraph of the "Discussion" section in our manuscript.

Overall, we really appreciate the comments from the reviewer. We have further revised our manuscript to make our claims clearer and more prominent. We hope that with our reply and explanations, we can clear up the misunderstandings and the reviewer can find our manuscript suitable for publication in Nature Communication.

Reviewers' Comments:

Reviewer #4:
Remarks to the Author:
I appreciate very much the detailed answers of the authors to my remarks. In particular, their explanations and corresponding revisions in the manuscript have significantly clarified the main claim for the reduction in transmitted information.

I do believe that the overall impact of the work is somewhat limited due the fact that no advantage in communication time has been shown and that the demonstrated advantage in transmitted information surpasses the best classical protocol and not the lower bound so there is essentially no new advantage shown but rather an improved performance with respect to previous results based on the idea of multiplexing that has also been previously suggested. Despite this, removing the need for demultiplexing and demonstrating the improved advantage in transmission efficiency over a long distance are important additional features of the presented protocol and worthy steps towards more complete implementations.

A final remark would be to please clarify in both the abstract and the introduction of the paper that no advantage in communication time has been shown in the proof-of-principle experiment and that the demonstrated advantage only concerns the transmitted information.

Reviewer #4 (Remarks to the Author):

I appreciate very much the detailed answers of the authors to my remarks. In particular, their explanations and corresponding revisions in the manuscript have significantly clarified the main claim for the reduction in transmitted information.

We thank the reviewer for taking time to review our manuscript again. We are so glad that our reply and our revised manuscript have removed the main concerns from the reviewer. We deeply appreciate all the insightful comments and suggestions from the reviewer, which have helped us improve our manuscript significantly. In the following, we address the remaining concerns from the reviewer.

I do believe that the overall impact of the work is somewhat limited due the fact that no advantage in communication time has been shown and that the demonstrated advantage in transmitted information surpasses the best classical protocol and not the lower bound so there is essentially no new advantage shown but rather an improved performance with respect to previous results based on the idea of multiplexing that has also been previously suggested.

We understand the reviewer's concern of the impact of our work. We would like to point out that, even though applying multiplexing to quantum fingerprinting has been theoretically explored in Ref. [35], no experimental implementations have been done before. In our work, we not only propose a fingerprinting protocol with the use of multiplexing and simultaneous detection, but we also report a proof-of-principle experimental demonstration to show the feasibility of our proposed protocol. We agree with the reviewer that an experimental demonstration showing both the reduction in communication time and less communication than the lower bound of classical fingerprinting would be more persuasive to demonstrate the significance of our protocol. We remark that our current experimental set-up can be modified to demonstrate the advantages of our protocol in both communication time and communication complexity given sufficient time and resources, using commercially available components.

Despite this, removing the need for demultiplexing and demonstrating the improved advantage in transmission efficiency over a long distance are important additional features of the presented protocol and worthy steps towards more complete implementations.

We appreciate the reviewer's recognition of these important features of our work. As mentioned by the reviewer, our proposal as well as our experimental demonstration of the WDM-CQF protocol provides an efficient technique for quantum fingerprinting implementation.

A final remark would be to please clarify in both the abstract and the introduction of the paper that no advantage in communication time has been shown in the proof-of-principle experiment and that the demonstrated advantage only concerns the transmitted information.

We thank the reviewer for this suggestion. We have revised our manuscript accordingly to make it clear that our experimental demonstration only concerns the reduction of the amount of transmitted information rather than communication time. Please see the revisions highlighted in red in the abstract and in the last paragraph in the introduction section.

In summary, we thank the reviewer again for the helpful comments. We hope that the reviewer will now find our paper suitable for immediate publication in Nature Communication.
